# Gendered networks and demand for an agricultural technology in India^☆^

**DOI:** 10.1016/j.worlddev.2025.107182

**Published:** 2026-01

**Authors:** Kajal Gulati, Nicholas Magnan, Travis J. Lybbert, David J. Spielman

**Affiliations:** a Michigan State University, East Lansing, MI, USA; b Colorado State University, Fort Collins, CO, USA; c University of California, Davis, CA, USA; d International Food Policy Research Institute, Washington D.C., USA

**Keywords:** Intrahousehold dynamics, Gender, Social networks, Technology adoption

## Abstract

Studies on social learning and technology adoption often only consider the networks of a single individual in a household as a source of information influencing agricultural production decisions. We test the validity of this assumption by examining the role of men’s and women’s social networks in the adoption of a novel water-saving technology, laser land leveling (LLL), in India. Using network data from men and women in the same household, we test the influence of being connected to an adopter on demand for LLL. We identify the causal gender-specific network effects using a field experiment that combines an auction with a lottery for the technology, making the presence of adopters in networks exogenous. The data reveal that men’s and women’s networks vary in size and show little overlap. We find that whereas household demand for LLL increases when men are linked to an LLL-adopting household, it decreases when the network linkages run through women. These gender-differentiated effects are concentrated in households where the woman’s opinion about the technology is valued by the man and in non-poor households. The results highlight that social learning may interact with the socio-demographic characteristics of households in myriad ways to influence household technology adoption decisions, and that agricultural-based information interventions ought to also consider how information gets used in the household.

## Highlights

• Women and men access distinct agricultural information networks.
• The effect of women’s and men’s networks on household’s technology demand differs.
• Women may have access to information, but it may not be valued within households.
• Studying social learning in heads’ networks may miss how households make decisions.

## 1 Introduction

Farmer learning about agricultural innovations is critical for improving farm productivity and rural incomes. Farmers often learn about agricultural technologies — their advantages, disadvantages, and how to use them — from other farmers. Because social networks are such an important source of information, an expansive literature has emerged on their role in agricultural technology adoption (Bandiera and Rasul, 2006, Beaman et al., 2021, BenYishay and Mobarak, 2019, Conley and Udry, 2010, Foster and Rosenzweig, 1995, Magnan et al., 2015). However, in many of these studies, only the networks of the household head or the primary decision-maker are considered as a source of information that influences the household’s production decisions. In the context of social learning, this operative assumption is highly restrictive not only because household members could draw on distinct sources of information, but also because production decisions could vary based on the gender of the decision-maker and on the prevalent intrahousehold dynamics (Beaman and Dillon, 2018, Gilligan et al., 2020, Gulati et al., 2024, Joshi et al., 2019).

In this study, we explore this knowledge gap by examining the role of social networks of men and women within the same household on their technology adoption decisions. Many agricultural decisions are made in consultation with household members, and information that individuals obtain from their own networks could be used in different ways in decision-making. Even though a given male or female household member might not contribute labor to an agricultural task, they could contribute valuable and complementary information that could influence the uptake of agricultural technologies, inputs, or practices that directly affect that task. This is especially relevant because men and women in the same household could have different expectations about the technology’s benefits, which may emerge from the information they acquire from their respective networks (Basu, 2006, Gilligan et al., 2020, Gulati et al., 2024, Joshi et al., 2019, Krishnapriya et al., 2024). As such, the fact that so much of the literature has considered only the role of the primary decision-maker’s networks on technology adoption decisions remains an important empirical shortcoming.

We examine these intrahousehold dynamics in social learning by focusing on the adoption of a novel water-saving technology in India. Laser land leveling (LLL) is employed in the land preparation phase of crop cultivation. Farmers generally level their land using a traditional leveler attached to a tractor to improve flood irrigation efficiency. LLL offers greater precision in leveling land as compared to a traditional leveler. Because most farmers irrigate their fields using diesel pumps, LLL saves money by reducing the duration of the diesel pump used and is the primary benefit of adoption (Jat et al., 2004, Lybbert et al., 2018). On average, LLL adoption lowers diesel costs by approximately 24 percent due to reduced groundwater pumping (Lybbert et al., 2018). While field trials have shown that LLL may enhance productivity by allowing for uniform application of inputs, farmer-level adoption data does not show any significant productivity gains (Jat et al., 2004, Lybbert et al., 2018). LLL involves the use of a tractor and sophisticated equipment, which farmers can access via hiring specialists who provide the service on a per-hour basis. At the time our study was conducted, the technology was novel to farmers at the study site. To introduce it and highlight its advantages over traditional leveling, a brief information session was held at the start of the intervention for both male and female household heads in the sample.

LLL exemplifies an agricultural technology that does not disproportionately affect gendered patterns of labor use or decision-making in land preparation. Its primary benefit — cost savings — accrues to the household as a whole.^1^ These benefits can vary depending on plot-specific factors such as slope, surface evenness, and soil texture. A household’s decision to adopt LLL depends on whether these perceived benefits outweigh the cost of hiring the service. Given this variability in benefits from LLL adoption, non-adopters’ primary learning about LLL within their networks centers on whether LLL delivers meaningful water savings and whether it provides other advantages, such as more uniform input application or yield improvements. Importantly, men and women may receive different types of information about LLL’s benefits or interpret the same information differently. As a result, their perceptions of the technology’s value — and thus their influence on the demand for LLL — can differ, even within the same household.

To explore the impact of social learning among men and women on household’s LLL demand, we designed a study with three key features. First, we measured the networks of women and men belonging to the same household using within-village sample matching. Using a village photo directory of the sampled household heads, we elicited men’s and women’s agricultural links to men and women residing in the sampled households. Second, we constructed a measure of household’s LLL demand before and after their exposure to the technology in social networks using a discretized incentive-compatible Becker–DeGroot–Marschak auction mechanism (Becker, Degroot, & Marschak, 1964). We thereby obtain a comparable measure of each sample household’s willingness-to-pay for LLL custom hire services, which are typically priced on a per-hour basis, at baseline — before they had any experience with LLL services — and a year later, after they had learned about LLL in their social networks. Third, to identify network effects, we conducted a randomized controlled trial (RCT) following the baseline auction, in which households were randomly assigned to receive LLL services. Households whose WTP was greater than or equal to the LLL bid price were randomly divided into two groups: one that received LLL services at the randomly drawn price, and one that did not. This randomization ensures that adopters in an individual’s social network are randomly assigned, conditional on the number of farmers who qualified for the RCT and the overall size of their agricultural networks. We use this random connection to an LLL adopter within men’s and women’s network to measure its effect on the household’s demand for LLL, after men and women had the opportunity to learn about LLL through these adopters’ experiences.

The study is part of a broader research program investigating various aspects of LLL adoption and diffusion. It is closely linked to two other studies within this program: Magnan et al. (2015) examine the influence of social learning in household heads’ networks on LLL demand, while Lybbert et al. (2018) analyze the determinants of LLL demand. Our study specifically focuses on exploring the interaction between gendered networks and household power dynamics, and how these factors influence LLL adoption.

Three main results emerge from this analysis. First, there is little overlap in the size and structure of men’s and women’s agricultural networks. On average, the size of women’s agricultural network — the number of connections a woman has in the village sample with whom she discusses agriculture — is larger than that of men’s agricultural network. In most households, either the man or the woman is linked to an adopting household in their respective agricultural network, but not both. This finding is consistent with previous research showing that women’s and men’s networks differ in size, structure, and the types of connections they include (Beaman and Dillon, 2018, Katungi et al., 2008, Meinzen-Dick et al., 2014) and Curran, Garip, Chung, and Tangchonlatip (2005). For example, in Uganda, Katungi et al. (2008) find that male- and female-headed households participate in different kinds of social capital groups, which serve distinct purposes for men and women. In India, a qualitative research shows that whereas women’s networks are primarily composed of family ties, men’s networks are more diverse, including farmers and friends (Padmaja, Bantilan, Parthasarathy, & Gandhi, 2006). More importantly, Beaman and Dillon (2018) demonstrate that social learning varies for men and women, due to differences in how information diffuses through male and female networks.

Second, we find the effects of men’s and women’s networks on household’s LLL demand are different: whereas household demand for LLL increases when a man is connected to at least one LLL adopter, it decreases when a woman has a link to a household that adopts LLL. These gender-differentiated network effects are conditional on the benefits that accrue to LLL adopters. When we disaggregate these links based on the water-saving benefits that accrue to adopters, we find that having a benefiting adopter in men’s network increases demand; in women’s networks, it has no effect. In contrast, having a non-benefiting adopter in men’s network has no effect on demand but, in women’s networks, it has a large negative influence. These results align with two recent studies that find similar differences in men’s and women’s demand for agricultural technologies when they receive information. Murphy, Roobroeck, Lee, and Thies (2020) find that women reduce their demand for organic inputs more than men when they receive information that organic input is not needed on their farm in Kenya. Gulati, Bauchet, Ricker-Gilbert, and Kane (2023) show that women in Senegal reduce their demand for food safety-enhancing agricultural technologies significantly more than men when they learn about aflatoxin prevalence in their village, even when their own crops have aflatoxin contamination. In contrast to our findings, in Ethiopia, Mekonnen, Gerber, and Matz (2018) show that adopters of row planting in a female’s network have a stronger influence than adopters in a male’s network on the household’s adoption decision. Even in contexts outside of agriculture, evidence shows that male and female peer effects influence behavior in different ways. Pakhtigian, Dickinson, Orgill-Meyer, and Pattanayak (2022) find that in India, women’s peer influence significantly increases latrine use among both women and men, whereas male peer influence has little to no effect.

Third, we find suggestive evidence that the socio-demographic characteristics of the household interact with men’s and women’s social learning in myriad ways, and that these dimensions are critical for understanding gendered network effects. When we disaggregate the sample based on whether the woman’s opinion is valued by the man in the household, we find that women’s network effects are present only in households where their opinion is valued. In households where the woman’s opinion is not valued, only the man’s network effects are positive, while the woman’s effects are non-existent. These gender-differentiated network effects are also observed only in non-poor households; in poor households, we find no economically meaningful network effects for either men or women. Another important dimension is the potential benefit the household stands to gain from adopting LLL. When we disaggregate the sample based on their level of diesel used for irrigation using diesel pumps, we find that men’s network effects are positive in both low- and high-diesel use households. In contrast, women’s network effects decrease demand in low-diesel-use households and have no effect in high-diesel-use households.

These findings are consistent with a growing body of literature that agricultural technology adoption is shaped by the preferences, constraints, and relative power of multiple household members (Ambler et al., 2021, BenYishay et al., 2020, Fehr et al., 2022, Gilligan et al., 2020, Gulati et al., 2024). A central finding of our study is the limited overlap between men’s and women’s agricultural networks and the distinct ways in which these networks influence household adoption decisions. In men’s networks, connections to adopters who report positive outcomes lead to increased household demand for the technology. In contrast, women’s networks often reduce demand, especially when their contacts report non-positive results. These contrasting patterns reveal that men and women interpret and transmit information differently: men’s networks may emphasize potential gains, while women’s networks may be more attuned to potential risks or losses. Together, these findings highlight the conceptual and econometric limitations of modeling social learning through a single household member, even if that person functions as the household head. Such an approach neglects the divergent influence of men’s and women’s networks. Moreover, the findings challenge the widely held assumption that women’s limited role in adoption decisions stems from informational gaps. Our results suggest that women often have access to relevant information — frequently as the only household member connected to adopters — but their influence over decisions could be constrained by limited agency. Thus, the key issue may not be access to information, but agency.

These results have implications for agricultural policy design and future research. From a policy perspective, a significant body of work has shown the existence of a gender gap in information and extension access (Peterman et al., 2011, Ragasa et al., 2013). Our results, together with other emerging body of evidence, suggest that while having access to information for women and men is necessary, it is not a sufficient condition to ensure that it gets used in household decision-making (Jewitt, 2000). One also needs to be cognizant of how this information interacts with factors such as decision-making roles, wealth, and technology adoption benefits (Conlon et al., 2021, Fehr et al., 2022). Supply-side interventions geared towards just providing information to women may not yield significant success. Instead, interventions that empower women to acquire information they find relevant could be more beneficial because it implies that women are more likely to contribute to decision-making using that information.

From a research perspective, future work could aim to understand how households learn and how information gets used in agricultural decision-making. On one hand, literature has emerged on gender differences in social learning, and, on the other hand, literature has just begun to emerge on gender differences in information use within the household (Beaman and Dillon, 2018, BenYishay et al., 2020, Conlon et al., 2021, Fehr et al., 2022, Gulati et al., 2023, Murphy et al., 2020, Posey and Magnan, 2023). Our research shows that social learning and gendered network effects are related to the socio-demographic characteristics of the household. Further research on the determinants of information use within households and the influence of gendered social learning on household decision-making is essential for understanding the efficiency of household decisions.

## 2 Conceptual framework

We depict social learning in the household using a simple framework. Assume the household consists of a man (m) and a woman (w), j∈{m,w}, with the man as the household head. We assume information, I, that the household has about using a new technology is an input in crop production. Specifically, the information augments the use of other inputs and improves the efficiency of their application (Conley & Udry, 2010). We also assume that the household members have no prior experience about using the technology and learn about the technology’s heterogeneous benefits from others. Let $I_{w}$ represent the set of individuals adopting a new technology in woman’s social network and $I_{m}$ represent the set of adopters in the man’s network. The information obtained from these adopters in j’s network, $I_{j}$, is pooled using a concave mapping function, $f_{j}\left(I_{j}\right)$, which enables the man and the woman to assess the relevance, quality, and usefulness of the information acquired from the adopters. The man and the woman may differ not only in the size and structure of $I_{j}$, but also in how this information is mapped, $f_{j}\left(\cdot \right)$.

Next, we model the pooling of men’s and women’s information as an intra-household bargaining process to form the overall information (I) the household uses to make crop production decisions. Specifically, we represent I as a weighted sum of the information that the man and the woman receive from adopters in their networks.

(1) $$I=\lambda \cdot f_{m}\left(I_{m}\right)+\left(1-\lambda \right)\cdot f_{w}\left(I_{w}\right)$$

Here λ∈[0,1] and represents the weight that is put on man’s information and 1−λ is the weight on woman’s information. The weight placed on woman’s information could depend on many factors, such as her bargaining power, involvement in agriculture, the relative size of her network, education, access to extension, and the overall quality of information that she receives (Frankenberg & Maluccio, 2003).

Most empirical studies on social learning in agriculture implicitly assume that a single individual (most likely, the household head) is the sole learner and sole decision-maker in the household. That is, implicitly, either λ=1 or $I_{w}=0$. In the empirical analysis that follows, we capture the agricultural networks of the man and the woman belonging to the same household and measure the degree of overlap in their network links. Descriptively, we aim to establish whether $I_{m}=I_{w}$. Next, we examine whether the woman’s information set via her social networks is considered in the household’s technology adoption decision. Although our research design does not allow us to estimate λ directly, we indirectly test the hypothesis of whether only the household head’s links are relevant for learning about new agricultural technologies. If we fail to reject the hypothesis that a woman’s information links have no effect on the household’s technology adoption decision, it indirectly suggests that we fail to reject the null hypothesis that λ=1. Finally, we examine whether the man and the woman respond in the same manner to receiving information from adopters in their network. That is, we measure whether $f_{m}\left(\cdot \right)$ and $f_{w}\left(\cdot \right)$ have a similar effect on the household’s technology adoption decision.

For tractability and to align the theoretical foundation with the empirical work, we restrict $f_{j}\left(\cdot \right)$ to depend only on the information acquired through an individual member’s network. In reality, it could be a function of other household member’s information mapping. That is, the woman’s mapping function could be represented as $f_{w}\left(f_{m}\left(I_{m}\right),I_{w}\right)$ and vice versa. This suggests that social learning within households could be more complex, and is described in greater detail in Appendix A.

## 3 Research design

This study is part of a broader research project focused on accelerating the adoption of LLL in the eastern part of the Indo-Gangetic Plains.^2^^,^^3^ At the time of this study, LLL was being increasingly adopted elsewhere in India but was largely unknown in our study region. LLL improves the precision of input application, especially irrigation water, fertilizer, and chemicals (Jat et al., 2004). Without precise leveling of the field, approximately 10–25 percent additional irrigation water gets used, which can be particularly expensive for farmers using diesel pumps (Jat et al., 2004). Although farmers use a traditional leveler attached to a tractor to level their fields during the land preparation phase, the leveling precision is between 4–15 cm.^4^ In contrast, LLL achieves a precision of close to 1 cm (Jat et al., 2004). This precision reduces irrigation water use by 10–30 percent, and evidence from this project shows a 24 percent reduction in water use and INR 350 per acre in diesel savings in the first year (Lybbert et al., 2018).^5^ These benefits vary with plot size, slope, and household wealth. Evidence on yield, nitrogen efficiency, and fertilizer use is mixed (Jat et al., 2004, Lybbert et al., 2018). Custom hiring is the most common way to adopt, and farmers pay on an hourly basis. Both LLL and traditional leveling typically require 3–8 h per acre, depending on the plot’s slope and degree of unevenness. LLL is not necessarily labor-saving compared to traditional leveling as farmers must be present in the field during LLL service delivery.

Against this backdrop, our study examines whether the social networks of household members not directly targeted with information, particularly women, affect household technology adoption decisions. This section provides an overview of the study’s sample, the LLL intervention, and the data collection process. The sampling subsection describes village and household selection criteria. The intervention subsection describes how initial LLL adopters were selected and how demand was measured before and after the intervention. The data collection subsection describes the survey and network data used in our analysis.

### 3.1 Sample

The study was conducted in three districts of EUP, selected to represent heterogeneity in agricultural productivity. In the selected districts, LLL was a new technology, and had been introduced in a few villages a year prior to our intervention.^6^ In each district, we randomly selected four villages. For each of these 12 selected villages, we randomly chose another pair village within a five-kilometer radius, yielding a total of 24 sample villages. This pairing strategy allowed us to examine cross-village network connections. Villages were excluded based on three criteria. First, we excluded villages with a population exceeding 400 households (above the 90th percentile in the study districts) to ensure full household enumeration and adequate within-sample network links. Second, we excluded villages where the rice-wheat (or a two crop) cultivation system did not exist because of flooding. Because our objective was to measure the extent of water saving accruing to LLL adopters, we wanted to capture the benefits for both rice and wheat cultivation. Last, we excluded villages which were within a 10 kilometer radius of any prior LLL exposure, using a complete list of villages with any pre-intervention LLL introduction.

In each sample village, we randomly selected approximately 20 households cultivating rice and wheat and with at least one plot greater than 0.2 acres, the minimum size required for LLL operation. Although the initial intervention involved 478 farmers, two forms of attrition reduced the sample size. First, a subset of households was absent during the second auction round. Second, in some households, we could not collect data on women’s networks and household decision-making.^7^ The final sample for this study comprises 380 households, consisting of 305 non-adopters and 75 adopters. Approximately, 82 percent of the sample households are headed by men. Appendix B shows the sample used for this study in relation to the broader research project.

### 3.2 Demand elicitation and LLL intervention

**Fig. 1:**
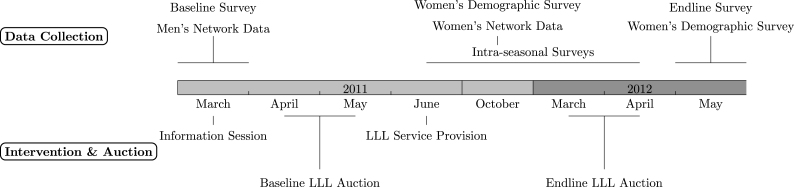
Timeline of data collection and field experiment.

The main intervention involved providing LLL services to randomly selected farmers and evaluating the change in demand for LLL services one year after learning about the technology from LLL adopters. A timeline for the intervention and data collection is shown in Fig. 1. At baseline, only five households were aware of LLL. To introduce the technology, the research team held brief (approximately one-hour) village-level information sessions with all sample household heads. In the session, we began by verbally introducing LLL and discussing its merits compared to traditional leveling. This was followed by a video demonstrating how the technology works in the field. The session culminated with a service provider answering questions about LLL custom-hire service provision. Farmers were informed that, like traditional leveling, the benefits of LLL would vary for individual farmers and depend on plot characteristics such as slope and levelness, but that LLL offers greater precision. We emphasized that we were not commercially promoting the technology to them. We delivered consistent messaging about the price of LLL rental services and informed the farmers that in recent years, the rental price of LLL had ranged from INR 400–800 in other parts of the state where LLL was more prevalent, though prices were undefined locally due to the absence of an existing market. At the end of the session, we distributed an LLL information card and informed the participants that the research team would return in a few days at which time the farmers would have an opportunity to custom hire LLL services. In the days following the information session, enumerators collected baseline and network data from household heads.

The research team returned a few days after the household data collection to conduct the baseline experimental auction organized at the same time for all sample households in the village. We elicited the willingness-to-pay using a Becker–DeGroot–Marschak auction mechanism for up to three plots identified by the participants as most promising for LLL (Becker et al., 1964).^8^ Appendix C illustrates how we collected the WTP for each of the farmer’s plots.^9^ For each plot, we asked the participants if they would be willing to custom hire LLL for a series of price points ranging from INR 250 to INR 800 per hour.^10^ For each plot, the bid ended once the participants reported that they would not be willing to pay a given price point, making the previous price their WTP for that plot. The auction was conducted in privacy, and we repeatedly reminded the participants that they should consider their own valuation for the technology when stating their valuation. After eliciting their WTP for each plot, we revealed the binding price to the participants (INR 250, 300, or 350 per hour). The auction procedure was incentive-compatible, as the binding price was unknown to the participants and they would have the opportunity to actually obtain LLL services if their WTP on any of the three plots was at least as high as the binding price. Prior to bidding for actual LLL services, farmers had the opportunity to practice the auction procedure by bidding on Indian sweets.

Farmers who had at least one plot with a bid greater than or equal to the binding price entered a 50–50 lottery, stratified based on the maximum WTP to determine the set of farmers who would then be able to actually obtain LLL services. This lottery is a critical dimension of the research procedures, enabling us to identify our network effects. Using the same notation as Magnan et al. (2015), the auction generated two groups of farmers: farmers who bid below the binding price (*auction losers*) and farmers who bid at or higher than the binding price (*auction winners*). These auction winners were eligible to enter the lottery, and hence, are termed as *qualifying farmers* in the remainder of the paper. The lottery allowed us to randomly divide *qualifying farmers* into two groups: those who won the lottery and were eligible to receive LLL services (*lottery winners*) and those who did not win the lottery (*lottery losers*). Whereas, on average, *auction winners/qualifying farmers* and *auction losers* are different groups, the *lottery winners* and *losers* are directly comparable by design.^11^

For the lottery winners, the research team coordinated the provision of LLL services on the plots that had a bid greater than or equal to the binding price during the land preparation phase of rice cultivation.^12^ Compliance with treatment was high, and 85 percent of auction winners obtained LLL services. These farmers comprise the set of LLL adopters in our sample. Further, no farmer other than the auction winners received LLL services. The most commonly cited reason for not obtaining services included heavy rainfall when the service provider was slated to visit the LLL village and the absence of the household when services were being delivered in the village.

A year later, after the rice and wheat cultivation seasons, we returned to the sample villages to conduct the endline LLL auction among the sample participants. The auction procedure was exactly like the baseline auction, with the exception that we did not implement a lottery.^13^ All farmers who bid higher than or equal to the binding price on at least one of their plots were eligible to receive LLL services. The endline auction allows us to measure the change in LLL demand after the adopters had an opportunity to learn about the benefits of the technology from their own use and non-adopters had the opportunity to learn about the benefits of the technology from adopters in their social network. The intervention culminated with LLL service delivery for eligible farmers during the second year’s rice land preparation phase.

### 3.3 Data collection

We collected the following data: (i) household’s socio-demographic information; (ii) plot-level input-use data for two agricultural seasons; (iii) men’s and women’s network data; and (iv) women’s contributions to agriculture and household decision-making. The timeline for these data collection activities is shown in Fig. 1. The household survey was collected for sample households in a village, in the days following the information session. We collected information about the household’s socio-demographic characteristics, asset ownership, and agricultural cultivation.

Along with collecting the socio-demographic information of households in this session, we collected household heads’ network data. Immediately after the information session, we photographed all the household heads in the sample to create a village photo directory. In the following days, we used these photos to collect baseline household and network data, eliciting each household heads’ agricultural, family, and friend links to other household heads in the village.^14^^,^^15^ To elicit agricultural links, we asked the farmer to list all the farmers in the photo with whom they discussed agriculture. To elicit family links, we asked the farmer to list all the farmers who were family. To list links to their friends, we asked the farmer to list all the farmers in the photo with whom they discussed general household matters, such as family and children’s issues. The network elicitation procedure accounted for the possibility that individuals could have multiple types of connections with the same individual — for example, a friend or a family member could also be an agricultural link. Because 83 percent of the household heads (from the entire sample of 478 farmers) were men, this round of data collection allows us to capture men’s networks. For our analysis, we define a link existing between farmer *i* and *j*, if farmer *i* reported being connected to farmer *j*, even if farmer *j* did not report such a link. This is valid because the way the question about discussing agriculture was framed reflects who farmer *i* considers a valuable source of information, and does not necessarily imply that farmer *j* would view farmer *i* the same way.

Women’s network data were collected a few months following the provision of LLL services in a village. We interviewed the primary women decision-makers living in the sub-sample of male-headed households (N = 310), along with the sample of women interviewed at baseline in female-headed households (N = 70) and elicited links to women living in the households of those shown in the photo directory.^16^ In our empirical analysis, we use men’s network data from 310 male-headed households and women’s network data elicited from 380 households. Although our project’s research agenda initially did not include capturing women’s social networks, our subsequent visits to these villages revealed that women possessed extensive knowledge about LLL. Many women reported acquiring this information from other women. Consequently, our team recognized that it would be remiss not to explore social learning among women in order to fully address the project’s research objectives.

We collected women’s networks using the same photos that we had used to capture the networks of household heads at baseline. Before eliciting networks, we tested if using the same photos would pose any risk to eliciting women’s networks. This involved examining whether women could recognize the household heads in the photos beyond those with whom they had agricultural, familial, or friendship ties. The pilot testing revealed that using the photo directory would not lead to any bias in data collection as women could easily identify the household heads and verified that they knew which women were living in those households. If women had links to sample households but failed to recognize them due to the use of the male household head’s photo, our estimates of women’s network effects would still be valid, provided that this lack of recognition is not systematic. This is because our identification strategy does not rely on capturing the full network structure. However, our estimates could be potentially biased if lack of recognition was related to the probability of adopting LLL. Although we piloted the photo directories with women from both wealthier and less wealthy households, we cannot rule out the possibility that this method introduced systematic bias in the elicitation of network data. As such, this remains a limitation of our network elicitation procedure.

Two particular aspects about women’s network data are worth noting. First, we only elicited women’s links to other women living in those households. To elicit the agricultural links, we asked each woman if they discussed agriculture with any women living in thehouseholds of those shown in the directory. In EUP, it is considered culturally inappropriate for women to talk to men outside their household, and even asking such a question from women would have been inappropriate.^17^ Second, the timing of women’s network data collection — after the first set of LLL adopters had used the technology on their fields — creates the potential for endogenous network formation if women sought information from LLL adopters who were not a part of their agricultural networks prior to LLL adoption and reported these adopters as belonging to their agricultural networks. We discuss this issue of potential endogeneity in women’s networks and its influence on capturing network effects in Section 6.2. Aside from the potential for endogenous network formation among women, other time-varying factors related to the different timing of network data collection for men and women do not pose a threat to our identification strategy. For example, if women formed new agricultural connections with other women during this period in ways unrelated to LLL, the likelihood of having an LLL adopter in their network would remain exogenous, conditional on network size and the presence of qualifying farmers within the network. Furthermore, other time-varying controls and socio-demographic characteristics collected for women after the LLL intervention are unlikely to pose a risk to the validity of our results.

Along with conducting women’s network data, we collected two rounds of data on women’s contributions to agriculture, their role in household decision-making, and whether they discussed LLL within their household and with others in their village (shown as “women’s demographic survey” in Fig. 1). One survey round was conducted when we elicited women’s network data and the other was implemented together with the endline input-use survey and the endline auction. These data allow us to understand how women’s networks interact with women’s power within the household and other socio-demographic characteristics to influence the household’s LLL demand.

To examine water savings and other benefits of LLL adoption, we collected detailed input use data at the plot level through intra-season surveys during both the rice and wheat cultivation seasons. These data allow us to quantify the benefits of LLL adoption in relation to farmers’ plot characteristics.

## 4 Data

### 4.1 Descriptive statistics

Table 1 provides a snapshot of the demographic and economic characteristics of men and women belonging to the sampled households. More than half of our sample belongs to the lower caste and about 65 percent fall below the average of the wealth index constructed from our data.^18^ About 82 percent of the households are headed by men, and 83 percent of the women and men in sampled households are related by marriage.^19^ Each household reported cultivating approximately 1.3 acres of land. About 95 percent of the sample households reported using diesel pumps, and incurred an average diesel cost of INR 1657 per acre. The high usage of diesel pumps implies that the decision to adopt LLL — and potentially save money — would be relevant for the majority of households in the sample. Moreover, as shown in Lybbert et al. (2018), the average diesel savings from LLL adoption range between INR 309–405 per hour. Farmers generally level their land using a traditional leveler, which costs INR 150–250 per hour to rent. Whereas farmers were aware of the costs and benefits associated with traditional leveling, the extent of cost savings from LLL adoption or how these savings might vary depending on their plot characteristics was unknown to them at the onset of the intervention. The benefits associated with LLL adoption, compared to using a traditional leveler, constitute the main aspect of learning in social networks among men and women.

**Table 1:**
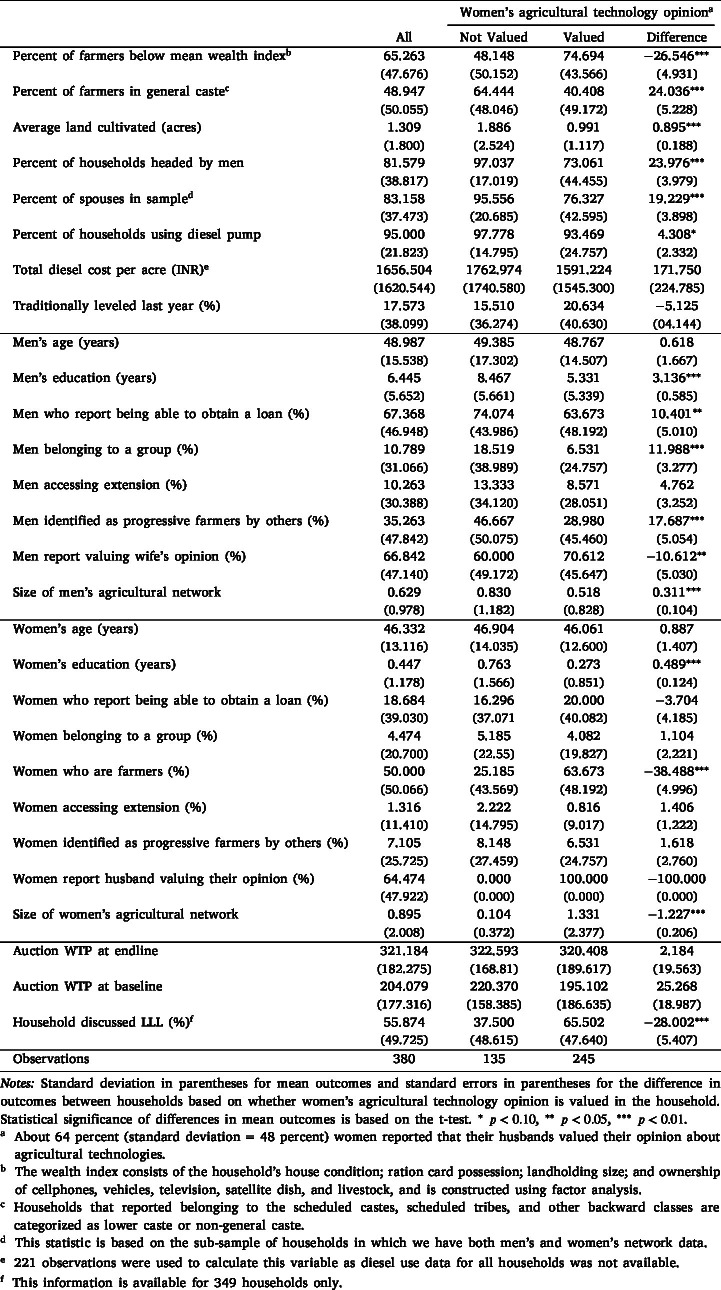
Summary statistics, disaggregated by whether women’s agricultural technology opinion is valued.

Comparing women and men belonging to the same household, we find men are roughly 3 years older with an average of almost 6 years of education more than women. All men in the sample engage in farming, whereas about 50 percent of the women surveyed engage in farm work. Although 35 percent of the men were identified by others in their network as progressive farmers, only 7 percent of women were reported as progressive farmers by other women. Women reported having access to lower levels of social and economic capital than men. About 67 percent of men reported being able to obtain a loan, 11 percent belonged to a formal or an informal group, and 10 percent had access to agricultural extension services. In contrast, only 19 percent women reported being able to get a loan, 4 percent belonged to a group, and about 1 percent had access to extension services.

We also examine differences based on whether the woman’s opinion on agricultural technologies is valued by the primary male- decisionmaker. About 64 percent women reported that the primary male decision-maker valued their opinion about agricultural technologies. Although Table 1 shows the summary statistics based on women’s report of whether their opinion about agricultural technologies is valued in the household, we elicited this information from men as well, by asking them if they valued their wives’ opinion about agriculture. On average, the same share of men reported valuing their wives’ opinion about agriculture, but there exists a disagreement in men’s and women’s reports. In 47 percent of households, both men and women reported that the woman’s opinion was valued by the man. In 14 percent of households, both men and women reported that the woman’s opinion was not valued by the man. In 39 percent of households, men and women gave different reports of whether the woman’s opinion was valued. The questions about whether a woman’s opinion is valued were asked differently from men and women.^20^ While the difference in question wording could account for the discrepancy, it could be that men and women vary in their perceptions of whether the woman’s opinion is valued, or men might have responded in a way that presents them as more supportive. While understanding the reasons behind this mismatch is beyond our scope, other studies have also found similar discrepancies in how women and men report decision-making and control over assets (Ambler et al., 2021). In the main empirical analysis, we show heterogeneity in gendered network effects based on both men’s and women’s reports.

We find important differences in the characteristics of households where the male decision-maker values and does not value the woman’s opinion about agricultural technologies. First, on average, households where women’s opinions are valued are poorer, belong to the lower caste, and cultivate less land, compared to households in which women’s opinions are not valued. Women whose opinion are valued are 38 percentage points more likely to be farmers compared to women whose opinions are not valued. These women also have slightly larger agricultural networks. Men who were reported as valuing women’s opinions are less educated, and are less likely to be able to obtain loans, belong to a group, or be identified as progressive farmers, compared to men who were reported as not valuing women’s opinions.

Next, we describe the key outcomes related to LLL demand and household discussion pertaining to LLL. The sample’s average willingness to pay for LLL increased from baseline to endline by INR 117. At baseline, 223 households qualified for the LLL lottery, and 96 won it to actually receive LLL custom-hire services. When LLL services were being delivered, a few households were not able to receive LLL due to logistical complications, and therefore 75 households form the set of initial LLL adopters (see Lybbert et al., 2018 for details). Moreover, in 56 percent of the households, men and women discussed LLL prior to the endline experimental auction. As expected, households in which women’s opinions are valued are 28 percentage points more likely to discuss LLL prior to the endline auction compared to households where their opinions are not valued.

### 4.2 Networks

Comparing men’s and women’s social networks, we find distinctive variation in network size and structure, as shown in Table 2. In a village with n sampled households, there are n⋅(n−1) possible links. In our sample, men could have 10,488 potential links and women could have 13,134 links. Women’s potential links are based on data from 380 women (310 from male-headed households and 70 from female-headed households), while men’s potential links are based on 310 men from male-headed households. This results in a difference in the total number of potential links for men and women. The actual proportion of friends, family, and agricultural links for men and women is low. Of the total links possible, men have 6 percent of those links as friends and 4 percent as agricultural connections. Of the total links possible for women, about 5 percent of those links are friends and agricultural ties. A total of 281 links in our sample were connections to family.

**Table 2:** Men’s and women’s social networks.

	Men’s total links^a^	Women’s total links^a^
	(Share of potential links)	(Share of potential links)
Friends	661 (0.063)	630 (.048)
Family	281 (0.027)	281 (0.021)
Agriculture	405 (0.039)	648 (0.049)
Agricultural links in same household	29 (0.003)	29 (0.002)

LLL adopter links	176 (.017)	233 (.018)
Lottery-winning agricultural links	190 (0.018)	237 (0.018)
Lottery-qualifying agricultural links	310 (0.030)	461 (0.035)
Friends who are agricultural links	121 (0.012)	380 (0.029)
Family links who are agricultural links	22 (0.002)	31 (0.002)
Links with whom discussed LLL at midline^b^	809 (0.077)	581 (0.044)
Links with whom discussed LLL at endline^b^	1586 (0.151)	779 (0.059)
Links where saw LLL operate	449 (0.043)	246 (0.019)

a In a village with n sample households, there are n⋅(n−1) possible unidirectional links. For men, 10,488 links and, for women, 13,134 links could potentially exist. We do not consider links between women and men due to cultural norms prohibiting women’s interactions with men outside their households. The actual total links that exist for each type (for example, friends, family, agriculture) are shown for men and women. The parentheses show these actual links in our sample as a share of all links possible.

b Midline refers to when women’s social network data were collected. Endline refers to network data collection when the endline experimental auction was conducted.

Women’s and men’s agricultural links also have very little overlap: only 29 links were to the same household.^21^ Women are also more likely to have agricultural connections with their friends as compared to men: 60 percent of women’s friends are also agricultural ties as compared to 18 percent of men’s friends who are agricultural connections. Another important dimension is the low degree of overlap in who brings information to the household about LLL: households in which both the woman and the man have an adopter in their agricultural network are few (only 6 households). Moreover, only men have a link to an LLL adopter in 67 households and only women have a link to an adopter in 79 households.

When we examine men’s and women’s agricultural links to auction-qualifying, lottery-winning, and LLL-adopting households, we find that the difference in probability of having an LLL adopter in men’s and women’s networks is not statistically significant. At the time women’s data were collected, men (women) reported discussing LLL with 8 (4) percent of their potential links, on average.

**Fig. 2:**
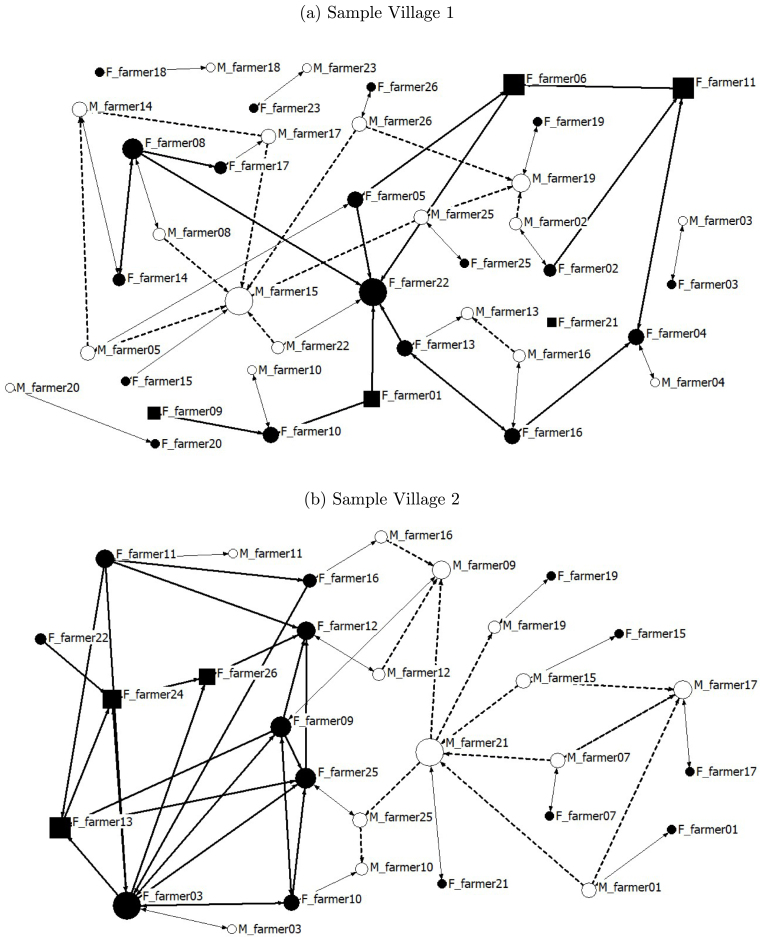
Agricultural networks of women and men in two sample villages. Notes: Each node represents a male or female farmer in the sample village. Male farmers are denoted by a white circle and female farmers are depicted as a solid black circle. The solid black squares indicate the household is headed by a female.The size of each node is scaled to a measure of the number of ties a node has relative to the total number of ties in the entire network. The thin lines between male and female nodes denote male and female farmers belonging to the same household. Thick lines (dashed for men and solid for women) indicate an agricultural link between two farmers, with single arrowheads representing a unidirectional relationship and double arrowheads representing a bidirectional relationship.

We further illustrate the differences in men’s and women’s agricultural networks in our sample using a network map. Fig. 2 represents the network map in a sample village (referred to as sample village 1) with actual agricultural links as reported by men and women. In this figure, each node represents a male or female farmer in the village sample. Male farmers are denoted by a white circle and labeled as M_farmer nodes, while female farmers are depicted by a solid black circle and labeled as F_farmer nodes. The solid black squares indicate that the household is headed by a female. The size of each node is scaled to a measure of the node’s degree of centrality, which is calculated as the number of ties a node has relative to the total number of ties in the entire network. The thin lines between male and female nodes denote male and female farmers belonging to the same household. Thick lines indicate an agricultural link between two farmers, with single arrowheads representing a unidirectional relationship in which farmer i reports farmer j as a member of their agricultural network (and not vice versa), and double arrowheads representing a bidirectional relationship between farmers i and j. Thick dashed lines represent relationships between male farmers, and thick solid lines denote relationships between female farmers.

As evident in the figure, there is very little overlap in men’s and women’s agricultural networks. In sample village 1, male farmer 15 is an important source of agricultural information. Six other men identify him as a person they talk to about agriculture, but he identifies no one in the sample that he talks to about agriculture. Note the absence of a corresponding role for the female in male farmer 15’s household. She has no network links to other women in the sample. In contrast, female farmer 22 plays a very central role in the female network, with five women reporting that they talk to her about agriculture. No men in the sample report talking about agriculture with male farmer 22, and he only stated talking to one man in the sample (the aforementioned male farmer 15). Also of note in Fig. 2 is that two households (4 and 10) are only connected to other households by female links.

The second panel in Fig. 2 shows the sampled network in another village (sample village 2). Again, there is little overlap between male and female networks. For some households (3, 11, and 12) in this village, women are much more connected, and for others (1, 17, and 21), men are more connected. We chose to depict this sample village because it illustrates the important role that female household heads can play in female networks. Here, female farmers 13, 24, and 26 are important sources of agricultural information as many women state talking to them about agriculture.

## 5 Empirical strategy

We test whether having an LLL adopter in a man’s and woman’s agricultural network affects the household’s demand for LLL. Identifying network effects is challenging due to the reflection problem: the difficulty in distinguishing if two individuals use the same technology because of learning or mimicry, or because they share correlated unobserved and observed characteristics that lead them to act similarly as those in their networks (Manski, 1993). Following strategies used in Kremer and Miguel, 2007, Magnan et al., 2015, and Oster and Thornton (2012), we leverage the randomized nature of the LLL lottery: conditional on the number of qualifying farmers in an individual’s network, the presence of an adopter is random.^22^ This randomization enables causal identification of network effects on LLL demand.

We estimate the effect of having an LLL adopter in men’s and women’s networks, controlling for: (1) the number of agricultural links who qualified for the LLL lottery, and (2) the size of their agricultural network. The main regression specification is:

(2) $$WTP_{2}=\beta_{0}+\beta_{1}\cdot adopt_{m}+\beta_{2}\cdot adopt_{w}+\beta_{3}\cdot qualify_{m}+\beta_{4}\cdot qualify_{w}+\beta_{5}\cdot netsize_{m}+\beta_{6}\cdot netsize_{w}+\beta_{7}\cdot WTP_{1}++X^{\prime }\alpha +\epsilon$$

Here, $WTP_{2}$ is the household’s endline willingness-to-pay for LLL. Variables $adopt_{m}$ and $adopt_{w}$ represent whether a man and a woman, respectively, have a link to an LLL-adopting household. We control for whether each is connected to one or more households that qualified for the lottery ($qualify_{m}$ and $qualify_{w}$), and for their agricultural network sizes ($netsize_{m}$ and $netsize_{w}$). For $qualify_{m}$ and $qualify_{w}$, we include indicators for being linked to one, two, three, and four or more individuals qualifying for the lottery, since the probability of having at least one adopter may increase with the number of links to households qualifying for the lottery. We measure $netsize_{m}$ and $netsize_{w}$ as the total number of agricultural links men and women have to households in the village sample. $WTP_{1}$ is baseline WTP, and ϵ is the stochastic error term.

We also control for individual and household characteristics (vector X), including the wealth index, whether the household head is male, the household caste, and the age and education of both the man and the woman.^23^ Because not all lottery winners adopted LLL, and no non-winners received it, we instrument links to an LLL adopter with links to households who won the LLL lottery.

Our key parameters of interest are $\beta_{1}$ and $\beta_{2}$, capturing the effect of men’s and women’s networks, respectively, on household’s demand for LLL. We test whether $\beta_{2}=0$; that is, whether a woman’s link to an adopter affects household demand. We also test whether $\beta_{1}=\beta_{2}$ to assess if the effects differ by gender. As a placebo, we re-estimate the model using baseline WTP as the outcome to test for pre-existing network effects, controlling for whether the household qualified for and won the LLL lottery, which may influence both the probability of having an LLL adopter and baseline WTP.

Beyond analyzing the aggregate gendered network effects, we disaggregate the results based on the nature and magnitude of benefits that adopters derive from LLL adoption. In our context, because LLL was available via custom-hire services, the primary learning in networks would likely be about the water-saving benefits of the technology. As discussed in Sections 3, 4, some adopters could have benefited from using the technology, as measured by the amount of water saved relative to LLL rental costs, and others may have not benefited significantly from LLL use. Men and women could learn about these differential benefits and respond differently to receiving such information. To test this aspect, we divide LLL adopters into two groups: adopters who saved water by using LLL (water saving) and those who did not experience any water-saving benefits (non-water-saving). Farmers who used 14 percent less water compared to the previous year are classified as water savers and farmers who did not save that much water are classified as non-water-saving households.^24^ We then estimate how women’s and men’s links to these two types of adopters in their agricultural networks influence LLL demand. Specifically, we estimate the following regression model.

(3) $$WTP_{2}=\beta_{0}+\beta_{1}\cdot {\left(adopt\text{\_}saver\right)}_{m}+\beta_{2}\cdot {\left(adopt\text{\_}nonsaver\right)}_{m}+\;\beta_{3}\cdot {\left(adopt\text{\_}saver\right)}_{w}$$

$$+\beta_{4}\cdot {\left(adopt\text{\_}nonsaver\right)}_{w}+\beta_{5}\cdot {\left(qualify\text{\_}saver\right)}_{m}+\;\beta_{6}\cdot {\left(qualify\text{\_}nonsaver\right)}_{m}+\;\beta_{7}\cdot {\left(qualify\text{\_}saver\right)}_{w}+\beta_{8}\cdot {\left(qualify\text{\_}nonsaver\right)}_{w}+\beta_{9}\cdot netsize_{m}+\;\beta_{10}\cdot netsize_{w}+\;\beta_{11}WTP_{1}+X^{\prime }\alpha +\epsilon$$

In Eq. (3), $adopt\text{\_}saver_{m}$ ($adopt\text{\_}nonsaver_{m}$) and $adopt\text{\_}saver_{w}$ ($adopt\text{\_}nonsaver_{w}$) represent whether men’s and women’s networks, respectively, have a water-saving (non-water-saving) LLL adopter. Further, we classify non-adopters into two groups based on the same threshold of water saving. To account for unobserved factors that could influence both household WTP and network links to water-saving and non-water-saving LLL adopters, we control for the number of each type of qualifying farmers. Variables $qualify\text{\_}saver_{m}$ ($qualify\text{\_}nonsaver_{m}$) and $qualify\text{\_}saver_{w}$ ($qualify\text{\_}nonsaver_{w}$), respectively, represent the number of water-saving (and non-water-saving) qualifying farmers men and women are connected to in their agricultural network.

Eq. (3) allows us to test two dimensions of gendered network effects. First, for both men and women, it allows us to understand if learning could be driving these network effects compared to other potential mechanisms. That is, if both $\beta_{1}$ and $\beta_{2}$ for men and ($\beta_{3}$ and $\beta_{4}$ for women) are positive, then it could be suggestive that farmers are likely mimicking other farmers by wanting their fields to be leveled.^25^ This is possible in our setting as LLL makes the fields look visibly smooth, compared to unleveled and traditionally-leveled fields, and other farmers may want the same for their fields. In such a scenario, the network effects we estimate may provide evidence of imitation rather than learning per se about the technology’s benefits relative to traditional leveling on farmer’s own fields. Because women are not directly involved in land preparation, if the direction of both $\beta_{3}$ and $\beta_{4}$ is the same, an alternative explanation is that women in LLL adopting households may not know about the degree of water-saving accruing to the household from LLL. In this case as well, if the point estimates are positive, it is indicative of imitation. If $\beta_{1}$ and $\beta_{3}$ are positive and $\beta_{2}$ and $\beta_{4}$ are negative, then the results provide evidence that learning could be driving the results instead of other characteristics or behavior of these network links (Manski, 1993).^26^ Second, the direction and magnitudes of $\beta_{1}$, $\beta_{2}$, $\beta_{3}$, and $\beta_{4}$ allow us to understand the differences between men’s and women’s network effects, conditional on receiving positive and negative information. That is, similar signals about the benefits of the technology may lead to differential impacts for women and men.

## 6 Results

### 6.1 Gendered network effects

To understand the influence of being linked to an LLL adopter in men’s and women’s agricultural networks on LLL demand, we estimate the regression model specified in Eq. (2) using the sample of non-adopters. The non-adopters comprise three groups, as detailed in Appendix B: (i) households that lost the auction (N = 157); (ii) households that lost the lottery (N = 127); and (iii) households that won the lottery but were not able to adopt LLL due to random reasons (N = 21). Table 3 shows our main results using endline WTP as the outcome variable. We find that the point estimates associated with men’s and women’s networks are statistically significant and economically meaningful, thereby suggesting that having an adopter in both men’s and women’s agricultural networks influences household demand. Being linked to an LLL adopter has differential effects for men and women: whereas being connected to an adopter increases household WTP by INR 88 for men, women’s network effects reduce household WTP by INR 65.^27^ The same table also shows the placebo test — using baseline WTP as an outcome variable — and shows that, as expected, the network effects associated with men’s and women’s links to adopters are not statistically significant or high in magnitude.

**Table 3:**
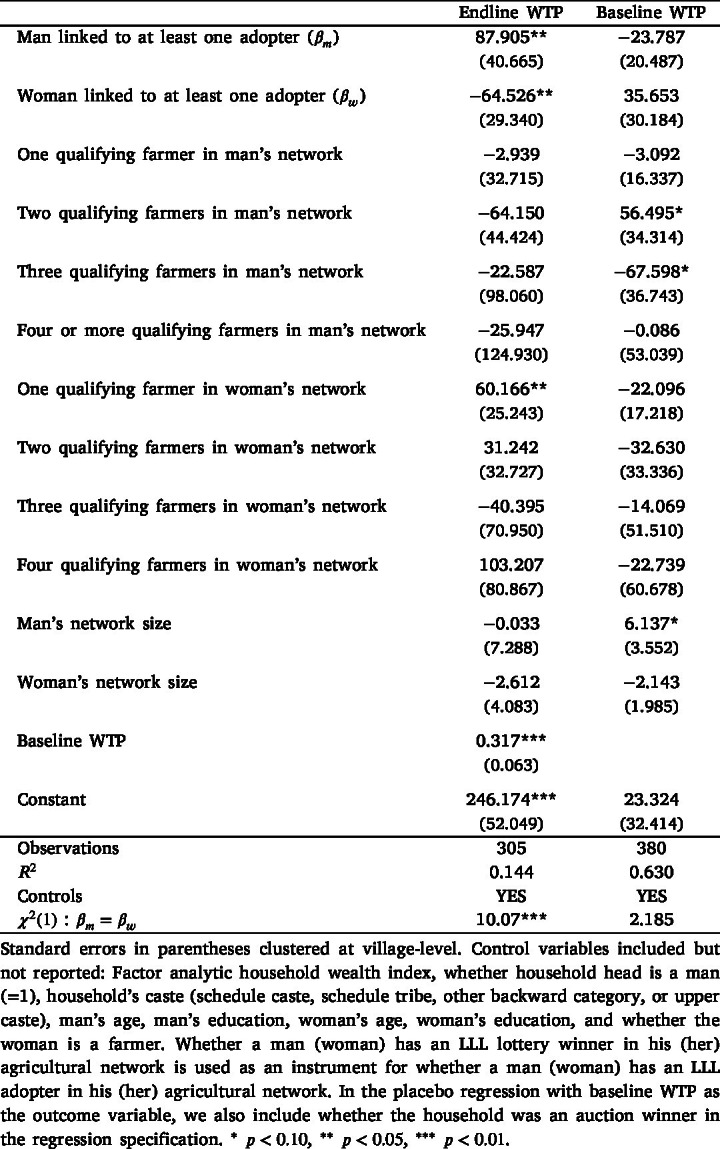
Men’s and women’s network effects on LLL willingness-to-pay .

To further explore these aggregate differences in men’s and women’s network effects, we classify LLL adopters into two groups based on the water-saving benefits that accrue to them, and estimate the regression model specified in Eq. (3). Table 4 shows the point estimates associated with men’s and women’s links to water-saving and non-water-saving LLL adopters, conditional on being connected to water-saving and non-water-saving qualifying farmers.^28^ We find that although being connected to a water-saving LLL adopter in men’s networks has a positive and statistically significant effect on household WTP (WTP increases by INR 146), there is no such effect for women. In contrast, being linked to a non-water-saving adopter has no meaningful effect for men but a large and negative network effect for women (WTP decreases by INR 97). These results suggest that men and women respond differently to receiving similar signals about the benefits of the technology. Moreover, the signs of the estimated coefficients (positive for when men and women are connected to a water-saving LLL adopter and vice versa) are suggestive of learning in networks, instead of them wanting to conform to other households adopting the technology.

**Table 4:**
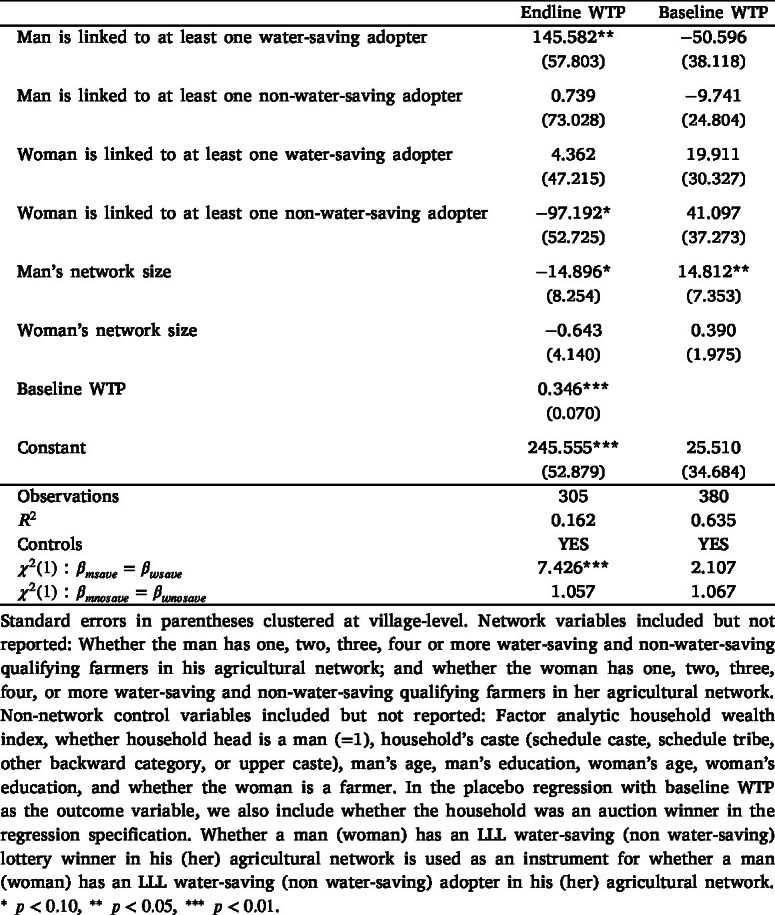
Network effects, based on water-saving benefits from adoption.

Another related aspect is that we should expect to see women’s network effects only among the sub-sample of households that discussed LLL prior to the auction, because only the household heads (primarily men in our sample) were invited to attend the endline auction and make the technology adoption decision for their household. Table E3 in Appendix E shows the relationship between LLL discussion and networks on household demand. We interact both men’s and women’s links to an LLL adopter with whether they discussed LLL prior to the endline auction. The results suggest that men’s network effects are similar in magnitude (approximately INR 85) but statistically insignificant, irrespective of whether the household discussed LLL. In contrast, women’s network effects differ based on whether the household discussed LLL. In households that discussed LLL before the endline auction, women’s network effects decrease WTP by INR 115 and are statistically significant. Women’s network effects are statistically insignificant and low in magnitude in households that did not discuss LLL. These results lend further support to the main results shown in Table 3.

Further, Table E4 in Appendix E presents network effect estimates for two groups of non-adopters: (i) lottery losers, who qualified for but did not win the lottery; and (ii) auction losers, who did not qualify for the lottery and had bid prices below the randomly drawn binding price. By design, these two groups differ in their ex-ante valuation of LLL (see Lybbert et al., 2018 for details). The sub-sample results indicate that the gendered network effects are primarily observed among auction losers. For lottery losers, the estimated effects for both men’s and women’s networks are small and statistically insignificant. Among auction losers, being connected to an adopter in men’s networks is associated with an increase in demand (though not statistically significant), while being linked to an adopter in women’s networks is associated with a decrease in demand. This is not surprising because auction losers had a lower baseline WTP valuation and could have benefited from learning about LLL in their networks. To understand how these gendered network effects manifest along the entire WTP distribution, we use dichotomous variables to measure if the endline WTP was greater than different WTP values, that is, whether $WTP_{2}\geq Price$, where price equals INR 250, 350, 450, and 600.^29^ WTP values above that range would reflect that farmers valued LLL and its benefits more than traditional leveling. Table E5 in Appendix E shows the network effects associated with WTP values being greater than the different price points. The direction of the network effects is positive for men and negative for women across all WTP values. These effects are most prominent, both in terms of magnitude and statistical significance, for WTP value being greater than or equal to INR 350, suggesting that network effects significantly contribute to increasing WTP for men for WTP values that are slightly greater than traditional leveler rental prices and significantly contribute to decreasing WTP for women at the same price points. The magnitude and statistical significance of these network effects reduces for higher WTP values.

### 6.2 Endogeneity

As noted earlier, the timing of network data collection for women — after selected farmers had adopted LLL — raises potential endogeneity concerns and threatens the identification of our results if women sought agricultural connections to LLL adopters and reported them as their agricultural links during data collection.^30^^,^^31^ To address these concerns, we explore several alternative network measures. First, we estimate network effects using women’s links to LLL adopters in their network of friends with whom they interact about general topics, not just agricultural topics. We elicited women’s links to friends by asking if they discussed family and children’s issues with women in sampled households in their village. It is highly unlikely that women reported LLL adopters as their friends if they had sought them because these women had adopted LLL, although we cannot fully reject this possibility. We also estimate network effects using women’s links to LLL adopters in their network of friends who are also in their agricultural network. This network — although more relevant for our analysis — could also be problematic for identifying network effects if women talked to friends who were LLL adopters and then reported them as their agricultural links.^32^

Table 5 shows the regression estimates using women’s links to their friends (column (1)) and to friends who are also their agricultural links (column (2)). The point estimate associated with being linked to an adopter in women’s friend network is negative and reduces household WTP by INR 51, but it is statistically insignificant. The point estimate associated with women being linked to an adopter in the subset of their friend’s network who are also their agricultural links reduces WTP by INR 79 and is statistically significant.^33^ Together, these results suggest that although the concerns regarding endogenous link formation cannot be entirely resolved, the magnitude and direction of our main results is robust to using alternative network measures and enhances our confidence in the main results.

**Table 5:**
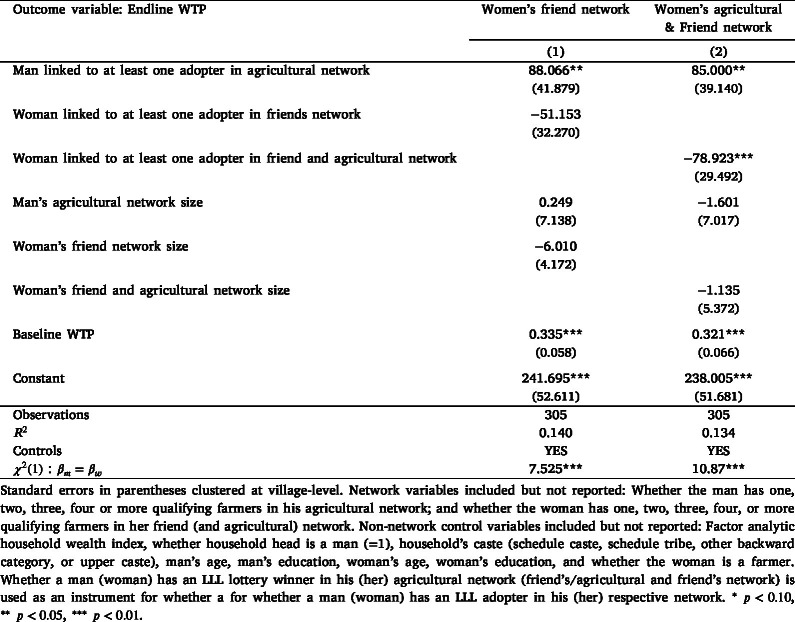
Gendered network effects, using women’s links to friends.

### 6.3 Heterogeneity

In this section, we examine heterogeneity in gender-differentiated network effects across three relevant dimensions, aiming to understand why men and women in the same household exhibit different network effects in technology adoption. This analysis is exploratory and suggestive: disaggregating networks reduces statistical power and these dimensions may directly affect the likelihood of being linked to an LLL adopter.

First, we consider technology attributes. Prior work suggests that differences in labor implications or perceived benefits may shape gendered preferences for technologies (Gulati et al., 2024, Joshi et al., 2019, Vemireddy and Choudhary, 2021). LLL is a precision technology that does not significantly alter labor allocation but provides its main benefit by conserving water, thereby reducing diesel use for irrigation. As such, household diesel expenses may not only directly influence the demand for LLL, but also the magnitude of gender-differentiated network effects. To explore this channel, we classify households as high or low diesel users, based on whether their per-acre diesel cost (which we only observe for a subset of households) is above or below the sample mean. We then estimate men’s and women’s network effects separately for each group (Panel A in Table 6; full regressions in Appendix F). Among low diesel-use households — where the potential primary benefit of LLL is less pronounced — network effects for both men and women are negative as expected, with women’s effects (−189 INR) more than twice as large and statistically significant compared to men’s (−78 INR). In contrast, in high diesel-use households, where the cost-saving benefits of LLL are more relevant, connections to adopters in men’s networks significantly increase household demand (359 INR), while women’s network connections have no effect. While based on a smaller sample, this pattern of results suggests an intriguing possible complementarity between these network effects: whereas women may only be able to influence household demand *downward* based on their networks if they are not poised to benefit much from LLL, men may be able to adjust household demand *upward* based on their networks when they stand to benefit more. Social learning in the case of LLL is thus associated with household decision-making that reflects expected benefits, but its expression differs between men and women.

**Table 6:**
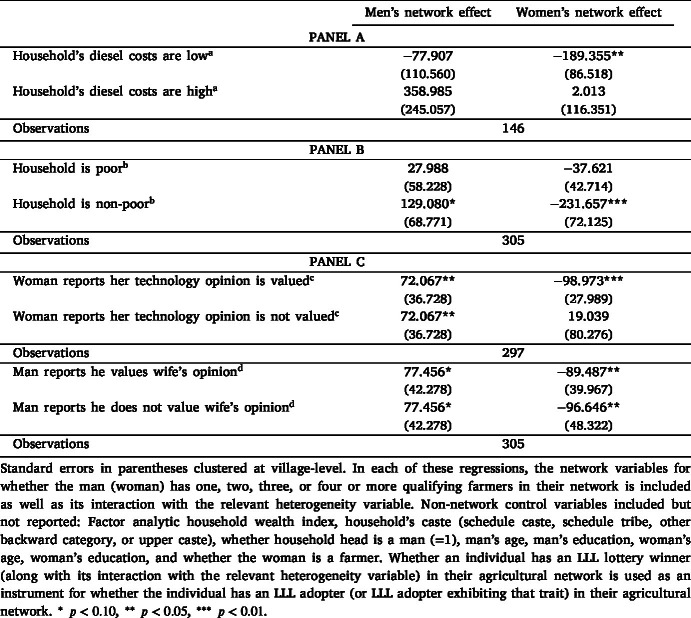
Heterogeneity in gendered network effects.

Second, men and women may perceive income constraints differently (Joshi et al., 2019). In our sample, reported loan eligibility differs by gender. We classify households as poor or non-poor based on the sample average wealth index and estimate network effects by wealth status (Panel B). In poor households, neither men’s nor women’s network connections influence WTP, suggesting binding income constraints. In contrast, in non-poor households, being connected to an adopter increases WTP by INR 129 for men and decreases WTP by INR 232 for women.

Third, the literature emphasizes the role of women’s voice in household decision-making, particularly for technology adoption (Gilligan et al., 2020, Gulati et al., 2024). When women have greater influence over agricultural decisions, they may be more likely to share and act on information acquired through their networks. To examine this, we disaggregate women’s network effects based on whether their opinions about agricultural technologies are valued in the household. This dimension is likely orthogonal to the probability of being linked to an adopter. We use two measures of this variable: women’s self-reports on whether they believe their opinion is valued and men’s reports on whether they value their wives’ opinion. These measures are interacted with women’s network connections to estimate heterogeneous effects (Panel C). When women report that their opinion is not valued, connections to adopters in their networks have no effect on household demand for LLL. In contrast, when their opinion is valued, these connections reduce household demand by INR 99, a statistically significant effect. However, results based on husbands’ reports show no difference in women’s network effects depending on whether women’s opinions are valued. It is plausible that men overstate the extent to which they involve their wives in decision-making (Ambler et al., 2021).

While we focus on three dimensions — technology attributes, household wealth, and women’s agency — other factors may drive gendered network effects. For example, gender differences in risk preferences or in the quality of information accessed through networks could matter (Beaman and Dillon, 2018, Magnan et al., 2020). Our findings highlight the complexity of technology adoption decisions and the importance of intersectionality. Though each dimension is analyzed separately to preserve statistical power, combinations likely matter. For instance, how do network effects differ for women in poor households who nonetheless bear high diesel costs or whose opinions are respected? While these results point to further avenues for research, they are also meaningful for policy design, especially in light of emerging evidence that women respond differently to agricultural information than men (Gulati et al., 2023, Murphy et al., 2020). These gendered patterns likely reflect a more complex set of factors than income or household decision-making differences alone.

### 6.4 Women’s social learning and household discussion

Next, we examine the relationship between men’s and women’s networks, household discussion of LLL, and women’s exposure to the technology. Table 7, column (1), presents the effect of men’s and women’s network variables on the household’s probability of discussing LLL before the endline auction. In addition to the network variables, we control for individual and household factors potentially associated with discussion. At the household level, we include caste, wealth index, and male headship. At the individual level, controls include men’s and women’s education, age, whether the respondent is considered a progressive farmer by peers, and reported loan eligibility. For women, we also control for participation in farm work.

**Table 7:**
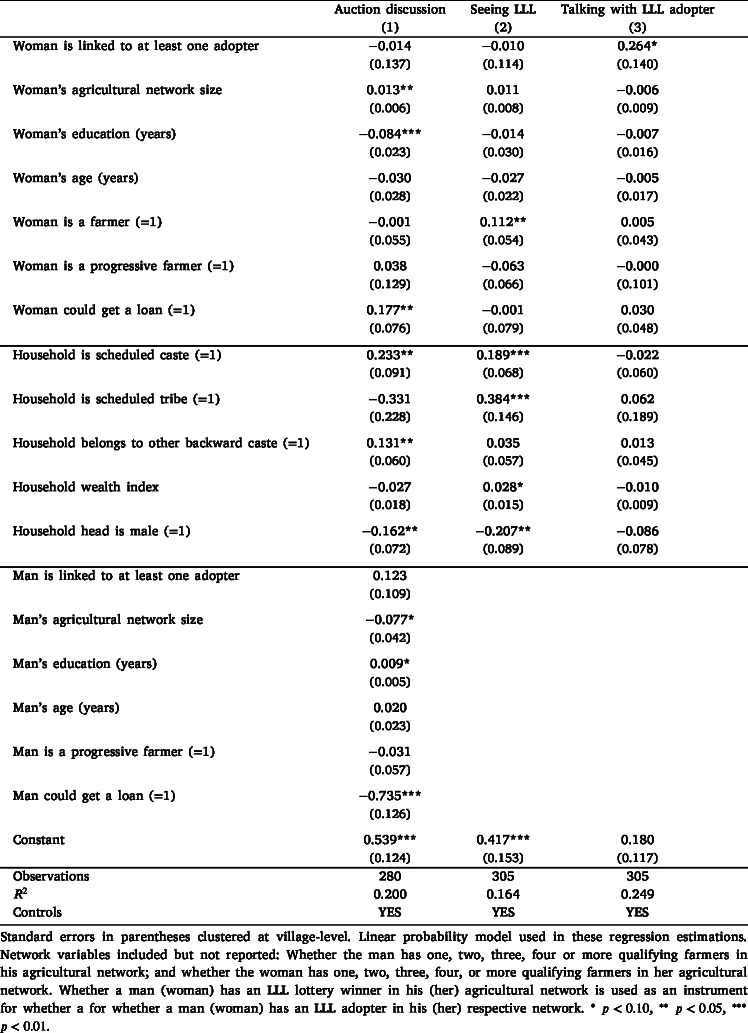
Women’s social learning about LLL.

The results show that whereas being linked to adopters is not significantly correlated with discussion, the size of agricultural networks is. Specifically, the probability of the household discussing LLL increases with the size of women’s networks and decreases with the size of men’s networks. One possible interpretation is that men with smaller networks may rely more on information acquired by women in their networks. Women’s ability to obtain a loan is also positively associated with the likelihood of household discussion, whereas the opposite is true for men. These findings suggest that women’s access to financial and social capital is correlated with their influence on household-level discussions about LLL. Additionally, an increase in women’s education levels decreases the likelihood of discussion, which aligns with existing evidence that more educated women in India are less likely to engage in agricultural activities (Srivastava & Srivastava, 2010).

Columns (2) and (3) of Table 7 examine whether being linked to an adopter affects women’s direct exposure to LLL — specifically, the probability of speaking to an adopter and seeing the technology in use. Being linked to an adopter increases the likelihood of a woman speaking to one by 26 percentage points. However, there is no statistically significant effect on the probability of seeing LLL operate in the field. This is consistent with context-specific constraints: mobility and time limitations often restrict women’s ability to visit adopters’ fields, and being connected to an adopter may not shift those constraints.

### 6.5 Limitations

While the study highlights important differences between men’s and women’s agricultural networks and how these differences influence household technology adoption decisions, we are unable to isolate the exact mechanisms driving the network effects. In particular, our data do not allow us to examine differences in the quality of information or the strength of signals transmitted through men’s and women’s networks.

Our network elicitation procedures raise two potential concerns for estimating the effect of women’s networks. First, because the data were collected after the initial set of households had adopted LLL, women may have subsequently formed links with those adopters, raising the risk of endogenous network formation. Second, despite significant pilot testing, field presence, and the use of relatively small-sized villages, we cannot fully mitigate the concern that using photo directories of male and female household heads to capture women’s links may have led to network measurement error. If women failed to identify female links because they did not recognize the male household head, our estimates should remain valid because our identification strategy does not rely on observing the complete network structure. However, women’s network effect estimates could be biased if non-recognition was systematically related to LLL adoption. For example, the probability of recognizing men in the photos may be correlated with the probability of adopting LLL based on certain traits — such as belonging to a particular caste, which could influence both recognition and LLL adoption.

More broadly, these findings are relevant to the adoption of agricultural technologies that do not significantly alter existing gender roles in agricultural production. In the case of LLL, adoption does not substantially affect farm labor use or change the roles that men and women perform on the farm. While our results may generalize to similar technologies, network effects could differ for technologies that impact labor demand, cropping patterns, or gender-specific labor roles. Additionally, our findings are relevant in joint agricultural production systems, where both men and women contribute to agricultural production, although in varying capacity, and men typically serve as the primary decision-makers. In our study region, despite men being the main farm managers, both family and hired women participate in agricultural operations to some extent. These findings may not extend to more agriculturally-advanced Indian states like Punjab and Haryana, where farms are larger and family women are often not involved in farm management at all, or to a West African context, where men and women often manage their own plots.

## 7 Conclusion

This study examines gendered differences in social networks within households and their implications for agricultural technology adoption. Using data from rural Uttar Pradesh, we show that men and women in the same household seek agricultural information from different sources and contribute unequally to adoption decisions through social learning. Connections to adopters in men’s networks increase household demand for LLL, while similar connections in women’s networks reduce it. This asymmetry reflects a deeper interplay between women’s influence over agricultural decisions, household wealth, and expected returns to adoption. Women’s network effects are concentrated in households where their opinions are valued, in non-poor households, and in those with below-average diesel use for irrigation.

Our findings contribute to a growing but limited literature on intra-household learning. Conlon et al. (2021) and Fehr et al. (2022) show that while wives do not differentiate between the information they receive from their husbands or themselves in making decisions, husbands discount wives’ information in Germany and India, respectively. Similarly, Posey and Magnan (2023) finds in Ghana that men selectively share information with spouses when it benefits them individually, while women share regardless of the information benefiting them individually or jointly. Consistent with this, we find women’s social learning shapes adoption only when their opinions are valued.

These findings highlight the need for more research on intra-household learning and its interaction with external information sources. While past studies link gendered preferences to labor-related technology impacts, our results show persistent differences in social learning even when labor effects are absent (Beaman and Dillon, 2018, Gulati et al., 2024, Joshi et al., 2019). As information access improves via digital technologies, understanding how households use such information is critical for effective agricultural policy.

## CRediT authorship contribution statement

**Kajal Gulati:** Writing – review & editing, Writing – original draft, Visualization, Project administration, Methodology, Investigation, Formal analysis, Data curation, Conceptualization. **Nicholas Magnan:** Writing – review & editing, Writing – original draft, Project administration, Methodology, Investigation, Funding acquisition, Formal analysis, Data curation, Conceptualization. **Travis J. Lybbert:** Writing – review & editing, Writing – original draft, Project administration, Methodology, Investigation, Funding acquisition, Conceptualization. **David J. Spielman:** Writing – review & editing, Writing – original draft, Visualization, Project administration, Methodology, Investigation, Funding acquisition, Conceptualization.

## Declaration of competing interest

The authors declare that they have no known competing financial interests or personal relationships that could have appeared to influence the work reported in this paper.

## Data Availability

Data will be made available on request.

## Appendix A Supplementary data

MMC S1 Online Appendix: Gendered Networks and Demand for an Agricultural Technology in India.

## Footnotes

1 In the study’s context, men in male-headed households are primarily responsible for implementing land preparation tasks and are the main decision-makers regarding which production technology to use for leveling their land.

2 All data collected as part of this research project are available at IFPRI (2017).

3 Two related published studies include Lybbert et al. (2018) and Magnan et al. (2015). Lybbert et al. (2018) show heterogeneity in water-saving benefits that accrue to farmers adopting LLL, and that these benefits are positively correlated with households’ *ex ante* LLL demand. Magnan et al. (2015) find that social learning about LLL occurs through household head networks and is influenced by the benefits adopters experience. Network data in Magnan et al. (2015) are limited to household heads — predominantly men — and, in female-headed households, only include ties to individuals in the photo directory.

4 At baseline, 32 percent of sample reported using a traditional leveler in the previous three years.

5 At the time of the study, 1 US Dollar was equal to 53 Indian Rupees (INR).

6 LLL had been introduced in a few villages from the following sources: (i) the broader project intervention, the Cereal Systems Initiative for South Asia (CSISA), that funded this study; (ii) the local agricultural research and extension unit of the state government, and (iii) one LLL unit owner who was working together with CSISA to provide custom hire services.

7 Common reasons for attrition included households not being present in the village during the auction implementation and the unavailability of the female decision-maker for an interview when enumerators visited.

8 The plots selected for the auction were larger than 0.2 acres and the farmers were planning on cultivating rice in the following season on them.

9 For the complete elicitation protocol, see Lybbert et al. (2018).

10 Leveling an acre typically takes 3–8 h, depending on the slope and evenness of the plot. Because of this variability, LLL services are usually priced on a per-hour basis. During the LLL auction, and before eliciting the WTP, the enumerators first helped each participant estimate how many hours it would take to level each of the three plots, based on the plot’s size, slope, unevenness, and the time it generally takes to level using traditional methods.

11 Detailed balance tests for these groups are presented in Lybbert et al. (2018) and suggest that lottery winners and losers have, on average, similar observable characteristics.

12 The research team partnered with a machinery company based in western Uttar Pradesh, which deployed four LLL units and service providers to eastern Uttar Pradesh before the rice planting season. Farmers in the sample villages had no other access to LLL services. At endline, the same company returned with four LLL units to provide services again. The key difference was that, unlike at baseline, service providers at endline could offer services to other farmers both within and outside the sample villages at prices they set themselves, but they were required to provide services to eligible sample farmers at the randomly-assigned binding prices.

13 We experienced some attrition during this phase: about 13 percent of the baseline auction households were unable to attend the endline auction due to random reasons. Even though we invited the household head to attend the auction, if they were unavailable, another key decision-making household member could attend the auction on their behalf. Unfortunately, we lack information on the gender or the relationship to the household head of the individual who attended the auction.

14 Given the short duration of the information session, it is highly unlikely that participants formed new network links while attending the session although we cannot fully ascertain this aspect.

15 Women appeared in the photo directory only if they were single heads of household. For these female heads, we initially collected data on their connections to other household heads, although these data are not used in this paper. Subsequently, when collecting network data for all women, we re-elicited the networks of female household heads. At that point, these female household heads reported their links either with other female household heads included in the photo directory or with women residing in the households of the male household heads shown in the directory.

16 About 83 percent of the women surveyed in male-headed households were wives of the household head, and the remainder were either the mother, sister, sister-in-law, mother-in-law, or “other”.

17 In the unlikely event that a woman did in fact speak with a man, she might not have reported this interaction due to concerns about adhering to culturally appropriate norms.

18 Households that reported belonging to the scheduled castes, scheduled tribes, and other backward classes are categorized as lower caste or non-general caste. In the empirical analysis, we use the different categories of caste for households. We construct the wealth index using factor analysis to represent the household’s asset ownership, based on the following variables: the household’s house condition; ration card possession; landholding size; and ownership of cellphones, vehicles, television, satellite dish, and livestock. The mean wealth index is 0 by construction.

19 This statistic is based on the sub-sample of households in which we have both men’s and women’s network data.

20 Women were asked a simple yes/no question: “Does your husband value your opinion about which technology to use?” For men, we asked a two-step question. First, they were asked, “Do you ever talk with your spouse about agricultural technologies and crop management?” If they responded yes, they were then asked: “How important do you consider your spouse’s opinion on agricultural technologies and crop management?” The response options were: not important, important, and very important.

21 Although there exists little overlap in men’s and women’s agricultural networks, we find similar factors are associated with the existence of an agricultural link among men and women. Appendix D presents these network formation results. For both women and men, belonging to the same family increases the probability of an agricultural link. Among men, those with smaller landholdings are more likely to be linked to individuals with larger landholdings. Poorer men are more likely to discuss agriculture with wealthier men. Belonging to the same caste increases the likelihood of forming a link among men. Men are also likely to form links with progressive male farmers. For women, being a farmer increases the likelihood of forming an agricultural link with other women, especially other female farmers. Women LLL adopters are also more likely to form a link to women in other non-adopting households. Importantly, for both men and women, plot-level characteristics such as soil type, surface evenness, and the amount of irrigation water used per acre are not significantly associated with link formation. This suggests that farmers are not necessarily linked to others with similar plot characteristics. As a result, individuals within a network may have different experiences with LLL. This opens up the possibility for farmers to learn about the technology’s heterogeneous impacts through their peers’ varied experiences.

22 We discuss potential endogeneity concerns for women’s network effects in Section 6.2.

23 We include whether the household belonged to the upper caste, scheduled caste, scheduled tribe, or other backward classes in our analysis.

24 The threshold of 14 percent water savings to classify households into water savers and non-water savers is obtained in the following way. The likely market price of LLL at the time the study was implemented would have been around 500 INR per hour. Not all farmers in our sample would accrue this level of benefit that would make it profitable to adopt LLL at this market price. Using pre- and post-intervention data on irrigation and diesel use, we estimate the proportion of farmers who would benefit from adopting LLL and the level of water saving they would have needed for LLL to be profitable at this market price. We calculate the level of water-saving under the assumption that water savings last for four years after adopting LLL, with no depreciation in the level of benefits. For more details, see Magnan et al. (2015).

25 In this context, mimicry does not necessarily entail conforming for the sake of social acceptance. Rather, it may stem from the belief that others possess more accurate information regarding the benefits of LLL. Thus, even in the absence of certainty about the specific advantages of LLL, a farmer may choose to imitate peers’ behavior as a rational strategy to enhance productivity.

26 Given the differential timing of network data collection for women, it is plausible that women intentionally formed connections with either water-saving or non-water-saving adopters. Although we cannot fully assuage this possibility, the share of women connected to a water-saving adopter and a non-water-saving adopter is similar.

27 Table E1 in Appendix E shows these gendered network effects are robust to restricting the sample to households in which the man and the woman are spouses, as opposed to being related in any other way. The table (column 2) presents results for a sub-sample that excludes female-headed households. These results indicate that, although network effects for women are negative, they are statistically insignificant and smaller in magnitude. This suggests that female-headed households are potentially an important factor shaping the network effects observed among women.

28 Full regression results showing the point estimates associated with the water-saving and non-water-saving qualifying farmers men and women are connected to are shown in Table E2 in Appendix E.

29 The average rental price of the traditional leveler during the study period was between INR 150–250.

30 Endogenous network formation in women’s agricultural networks is potentially the main concern in identifying network effects in our study’s context. Our network effect analysis is based on identifying the influence of an adopter in a network, and therefore, our results should not be biased because of partial network sampling. Our randomized intervention also allows us to avoid any biases arising from correlated shocks among individuals that share a link. For instance, if both individual i and j are linked and both are adversely affected by a decrease in the water table they share, then it could bias our network effects if they both have a high LLL demand, thereby making correlated shocks a part of the reflection problem. The probability of having an adopter in an individual’s network is random, conditional on the number of qualifying farmers in their agricultural network, and this mitigates any potential bias in network effects arising from correlated shocks.

31 As Appendix D suggests, we cannot reject the possibility of endogenous link formation based on examining the probability of two women sharing an agricultural link.

32 Another alternative would have been to use women’s links to LLL adopters in the subset of agricultural ties who are also family members. However, as shown in Table 2, family agricultural networks are sparse in our study sample and do not allow us to measure network effects in a robust manner.

33 Table E1 in Appendix E shows another robustness check. In our sample, 30 women reported having the same agricultural links as the women they had spoken to about LLL. If we remove these women from our sample, our network effects are stronger.
