# Supplementary material for: Gendered networks and demand for an agricultural technology in India
Source: World Dev. 2026 Jan;197:107182. doi: 10.1016/j.worlddev.2025.107182 (PMC12620961; doi:10.1016/j.worlddev.2025.107182)
Supplement: MMC S1 — Online Appendix: Gendered Networks and Demand for an Agricultural Technology in India. [file mmc1.pdf]

Online Appendix
Gendered Networks and Demand for an Agricultural Technology in India

Appendix A. Framework for Household Social Learning

We extend the model presented in the conceptual framework section. Let B_j represent the beliefs about the new agricultural technology held by household member j. These beliefs are shaped by the information each member obtains from their respective social networks, denoted as I_j, as well as the information acquired by the other household member. Let $f_j(I_j)$ represent a concave mapping function that aggregates the information available to each individual in their own networks. Based on these assumptions, we model the man's and the woman's belief formation as follows.

$$B_w = \theta_w \cdot f_w(I_w) + (1 - \theta_w) \cdot f_m(I_m) \quad (1)$$

$$B_m = \theta_m \cdot f_m(I_m) + (1 - \theta_m) \cdot f_w(I_w) \quad (2)$$

Equation 1 suggests that the woman's belief about the technology is shaped both by how she pools the information from her network, $f_w(\cdot)$, and by how the man maps the information from his network, $f_m(\cdot)$. The parameter θ_w captures the weight she assigns to information from her network, and $1-\theta_w$ represents the weight she places on information from the man's network. Comparing equations 1 and 2 suggests that the beliefs about the same technology can differ between the woman and the man due to three factors. First, they may access different networks, I_w and I_m. Second, they may process information differently, as depicted by their respective mapping functions, $f_w(\cdot)$ and $f_m(\cdot)$. Third, they may place different weights on the information received from each other, as represented by θ_w and θ_m.

Finally, these individual beliefs contribute to the household's adoption decision. Let I_h denote the composite information used in the decision-making process. We model I_h as the weighted average of each member's beliefs:

$$I_h = \lambda B_m + (1 - \lambda) B_w \quad (3)$$

In equation 3, λ represents the relative influence of the man's beliefs in the final household decision. It is important to distinguish λ from θ_j. Whereas θ_j represents how much weight each member places on the other's information when forming beliefs, λ determines whose beliefs dominate the household's technology adoption decision. Thus, λ reflects the division of decision-making roles in the household, while θ_j captures the internal information-sharing arrangements.

Appendix B. Study Sample

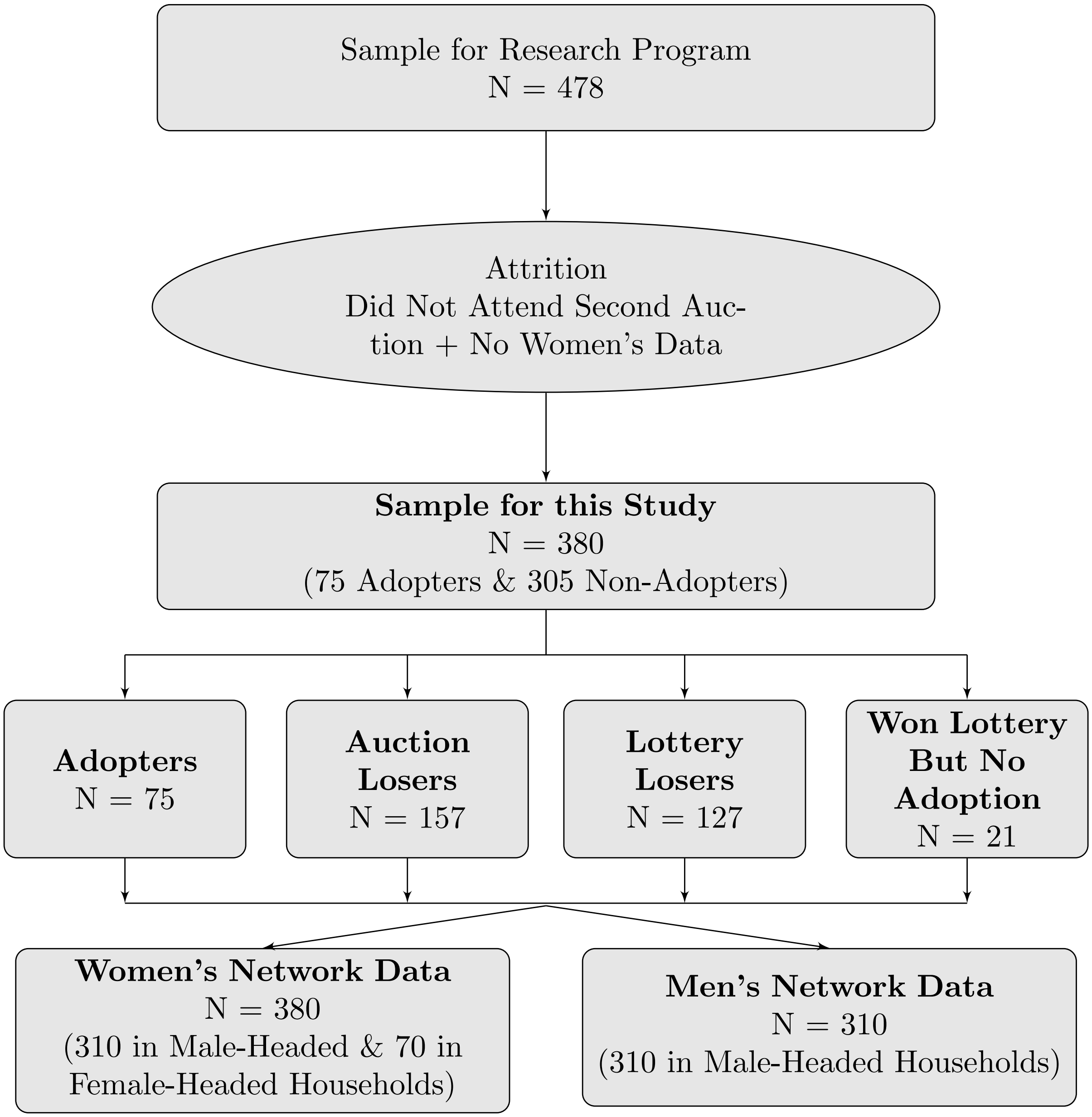

Appendix C. Willingness to Pay Elicitation

Household ID:__________ Are the plot names for this farmer correct? Y N Farmer's estimate of time to LLL						Real Price of LLL (INR/hour)									
	ISS plot name	*Auction plot name*	*Acres*	*LLL*	*Hrs*	*250*	*300*	*350*	*400*	*450*	*500*	*550*	*600*	*700*	*800*
**A**															
**B**															
**C**															

Appendix D. Network Formation

We examine the differences in characteristics that are associated with the formation of men's and women's agricultural links. To understand the determinants of link formation, we estimate the probability of the existence of a link between individuals i and j based on a vector of social and physical distance variables between i and j. We follow a similar estimation specification described in Fafchamps and Gubert (2007) and also used in other studies on network formation (Maertens and Barrett, 2013; Santos and Barrett, 2010).

The estimation strategy uses not only the absolute value of distance between two individuals but also the directionality of these distance variables. For continuous variables, such as age and years of education (represented by vector X^C), we use the absolute value of the difference between i and j and an interaction term between the absolute value of the difference and a binary variable for $i > j$. The parameter estimate for the standalone variable gives the effect of the distance on the individual with the lower value and the parameter estimate for the interacted variable indicates the effect of the distance on the individual with the higher value. For binary variables (denoted by X^D), such as household caste and being agriculturally progressive, we use binary variables for i but not j exhibiting the trait, for j but not i exhibiting the trait, and for both i and j exhibiting the trait. The omitted category represents neither i nor j exhibiting the trait. We also use the geographical distance, $Dist_{ijv}$, between the homes of the two households, and include whether i and j have family ties (fam_{ijv}). To account for similar sample but different population size within each village, we include a variable on village population in our estimation (Pop_v). To account for the correlation in the error term across pairs ij and ji, we employ dyadic standard errors calculated using the methodology proposed by Fafchamps and Gubert (2007).[1] Our overall regression specification is as follows.

$$\begin{aligned} Link_{ijv} = {} & \beta_{C1}|X^C_{iv} - X^C_{jv}| + \beta_{C2}|X^C_{iv} - X^C_{jv}| \cdot I(X^C_{iv} > X^C_{jv}) \\ & + \beta_{D1}I(X^D_{iv} = 1, X^D_{jv} = 0) + \beta_{D2}I(X^D_{iv} = 0, X^D_{jv} = 1) + \beta_{D3}I(X^D_{iv} = 1, X^D_{jv} = 1) \\ & + \beta_{fam}Fam_{ijv} + \beta_{dist}Dist_{ijv} + \beta_{pop}Pop_v + \epsilon_{ijv} \end{aligned} \tag{4}$$

Table D1 shows the estimation results for men's agricultural links formation. We find household characteristics, such as belonging to the same family, wealth, and land size are significantly correlated with the probability of an agricultural link among men. Men were about 6 percentage points more likely to talk about agriculture with men in the same family. Poorer men were more likely to report discussing agriculture with wealthier ones, whereas wealthier men were not significantly more likely to report talking to poorer ones (although

[1] To generate dyadic standard errors, we use the code written by Marcel Fafchamps and available on his webpage: https://web.stanford.edu/ fafchamp/resources.html.

the point estimate is negative). Similarly, men with smaller land size were more likely to report discussing agriculture with those with a larger land size, although those with a larger land size were not significantly more likely to discuss agriculture with those with a smaller land size. This asymmetry in conversations could reflect strategic link formation if men in wealthier households or those who cultivate more land have more or better information about agriculture than their poorer or small land-size counterparts.

Compared to agricultural information links between two non-progressive men, a progressive man was 2 percentage points more likely to discuss agriculture with a non-progressive man, a non-progressive man was 9 percentage points more likely to discuss agriculture with a progressive man, and a progressive man was 8 percentage points more likely to discuss agriculture with another progressive man. These findings indicate that progressive farmers are not only information providers, but also information seekers. We find no influence of differences in age, education, soil type, or geographical distance in men's agricultural link formation. We find no statistically significant likelihood of link formation between men based on whether they adopted LLL or qualified for the LLL lottery. These characteristics associated with men's agricultural links, especially based on who farmers identify as being a progressive farmer, lends support to the common extension strategy of reaching out to progressive farmers to promote new agricultural innovations, and does not support any potential network-induced information traps (Barrett and Carter, 2013).

The factors correlated with women's link formation are similar to those for men, but with a few differences as shown in Table D1. Family ties increase the probability of a network link by 5 percentage points. Unlike men, we find no influence of wealth on women's link formation, although women with smaller land size are less likely to have a link with those with a larger land size. We find that caste matters for women's link formation: a general caste women is 2 percentage points more likely to interact with a non-general caste women.

Compared to the probability of two non-progressive women discussing agriculture, two progressive women were 4 percentage points more likely to discuss agriculture. Unsurprisingly, women working on the farm were 3 percentage points more likely to discuss agriculture with a non-farmer compared to women who do not participate in farm work. Moreover, two women farmers were more 2 percentage points more likely to discuss agriculture as compared to two women who are not farmers. As the village population increases, the likelihood of women's agricultural link reduces.

Unlike men's network formation, women in non-adopting households are 2 percentage points more likely to have an agricultural link with women in adopting households. Because we collected women's network data after the baseline LLL auction, it is possible that women's agricultural information links are endogenous to technology adoption.

Table D1: Men's and Women's Network Formation

Dependent variable: Agricultural link	Men Coefficient	Men Marginal Effects	Women Coefficient	Women Marginal Effects
Link j is family	1.622***	0.049***	1.444***	0.051***
	(0.327)	(0.009)	(0.373)	(0.012)
$\|\Delta age\|$ (decades)	-0.035	-0.001	-0.144	-0.005
	(0.057)	(0.002)	(0.093)	(0.003)
$\|\Delta age\|$ if $age_i > age_j$	-0.031	-0.001	0.071	0.003
	(0.094)	(0.002)	(0.090)	(0.003)
$\|\Delta edu\|$ (years of schooling)	-0.029	-0.001	-0.071	-0.002
	(0.019)	(0.001)	(0.101)	(0.003)
$\|\Delta edu\|$ if $edu_i > edu_j$	0.020	0.001	-0.126	-0.004
	(0.029)	(0.001)	(0.193)	(0.004)
$\|\Delta wealth\|$ (wealth index)	0.288***	0.009***	-0.087	-0.003
	(0.078)	(0.002)	(0.142)	(0.004)
$\|\Delta wealth\|$ if $wealth_i > wealth_j$	-0.203	-0.006*	-0.206	-0.007
	(0.120)	(0.003)	(0.195)	(0.006)
$\|\Delta land\|$ (acres)	0.069***	0.002**	-0.118	-0.004
	(0.020)	(0.001)	(0.083)	(0.003)
$\|\Delta land\|$ if $land_i > land_j$	-0.015	-0.000	-0.145	-0.005
	(0.042)	(0.001)	(0.118)	(0.004)
$\|\Delta plot\ slope\|$	-0.034	-0.001	0.116	0.004
	(0.132)	(0.003)	(0.107)	(0.003)
$\|\Delta plot\ slope\|$ if $slope_i > slope_j$	-0.176	-0.005	0.001	0.000
	(0.187)	(0.004)	(0.094)	(0.003)
i has heavy soil, j has light soil	0.138	0.004	-0.023	-0.001
	(0.219)	(0.006)	(0.233)	(0.006)
j has heavy soil, i has light soil	-0.259	-0.008	-0.221	-0.008
	(0.284)	(0.007)	(0.291)	(0.007)
Both have heavy soil	-0.010	-0.000	-0.243	-0.009
	(0.289)	(0.006)	(0.295)	(0.007)
i general caste, j not	0.015	0.000	0.446	0.016
	(0.284)	(0.009)	(0.250)	(0.008)
j general caste, i not	-0.193	-0.006	0.300	0.011
	(0.300)	(0.008)	(0.240)	(0.007)
Both general caste	0.551*	0.017*	0.097	0.003
	(0.273)	(0.008)	(0.358)	(0.008)
i household head, j not			-0.054	-0.002
			(0.269)	(0.008)
j household head, i not			0.095	0.003
			(0.299)	(0.007)
Both household head			0.811	0.029
			(0.592)	(0.011)
i farmer, j not			0.848**	0.030**
			(0.294)	(0.008)
j farmer, i not			-0.053	-0.002
			(0.310)	(0.009)
Both farmer			0.750*	0.026*
			(0.366)	(0.008)
i progressive farmer, j not	0.964*	0.029*	0.166	0.006
	(0.442)	(0.011)	(0.327)	(0.010)
j progressive, i not	2.636***	0.080***	0.354	0.012
	(0.347)	(0.010)	(0.302)	(0.009)
Both progressive	2.522***	0.076***	0.904	0.032
	(0.375)	(0.011)	(0.512)	(0.017)
i adopted LLL, j not	0.192	0.006	0.501	0.018
	(0.250)	(0.007)	(0.274)	(0.008)
j adopted LLL, i not	0.017	0.001	0.527*	0.019*
	(0.297)	(0.007)	(0.247)	(0.007)
Both adopted LLL	0.484	0.015	0.744	0.026
	(0.300)	(0.009)	(0.437)	(0.011)
i qualified for LLL lottery, j not	-0.231	-0.007	0.007	0.000
	(0.227)	(0.009)	(0.325)	(0.009)
j qualified for LLL lottery, i not	-0.165	-0.005	0.114	0.004
	(0.263)	(0.008)	(0.339)	(0.008)
Both qualified for LLL lottery	-0.025	-0.001	0.122	0.004
	(0.300)	(0.008)	(0.402)	(0.008)
Household distance (km.)	0.035	0.001	0.347	0.012
	(0.212)	(0.007)	(0.208)	(0.006)
Village population	-3.361	-0.102	-2.835	-0.100
	(2.973)	(0.060)	(3.594)	(0.064)
Constant	-5.006***		-3.625***	
	(0.521)		(0.622)	
Observations	6154	6154	6154	6154

Logit regression model used for estimating probability of agricultural links. Dyadic standard errors shown in parentheses in columns (1) and (3). Omitted dummy variable for dichotomous variables is neither i nor j exhibiting the trait.* $p < 0.05$, ** $p < 0.01$, *** $p < 0.001$.

Appendix E. Robustness Checks

Table E1: Gendered Network Effects, Based on Sub-Samples

Outcome variable: Endline WTP	Husband-Wife<br>(1)	Male-Headed<br>(2)	Without Link Overlap<br>(3)
Man linked to at least one adopter	104.438***	96.132**	77.223*
	(40.105)	(43.959)	(43.349)
Woman linked to at least one adopter	-71.206**	-52.803	-113.727***
	(29.595)	(45.680)	(39.173)
One qualifying farmer in man's network	-27.291	-6.666	-3.762
	(26.984)	(31.768)	(34.850)
Two qualifying farmers in man's network	-82.590	-69.012	-53.800
	(51.303)	(49.172)	(44.320)
Three qualifying farmers in man's network	-38.449	-39.198	-41.242
	(105.732)	(100.463)	(113.847)
Four or more qualifying farmers in man's network	-66.173	-58.013	-58.601
	(148.119)	(144.161)	(126.258)
One qualifying farmer in woman's network	62.873**	52.828	54.405*
	(25.589)	(33.724)	(29.314)
Two qualifying farmers in woman's network	54.644*	6.884	79.839*
	(31.644)	(44.375)	(46.182)
Three qualifying farmers in woman's network	-49.826	-67.257	7.205
	(68.375)	(70.869)	(82.451)
Four or more qualifying farmers in woman's network	135.920	109.546	202.383**
	(85.986)	(86.951)	(83.280)
Man's network size	1.209	1.933	1.748
	(8.540)	(8.406)	(7.611)
Woman's network size	-3.972	-4.294	-3.830
	(4.554)	(4.500)	(4.457)
Baseline WTP	0.236***	0.262***	0.299***
	(0.073)	(0.073)	(0.077)
Constant	257.976***	254.827***	250.186***
	(69.376)	(54.984)	(59.566)
Observations	251	243	275
R^2	0.098	0.112	0.143
Controls	YES	YES	YES
$\chi^2(1) : \beta_m = \beta_w$	13.99***	7.692***	8.073***

Standard errors in parentheses clustered at village-level. The regression in Column (1) uses the sample of spouses (as opposed to also including men and women related in other ways in the household). The regression in Column (2) uses the sample of women and men in male-headed households and excludes female-headed households. The regression in Column (3) are based on the sample of households after excluding households in which women's agricultural networks and the links with whom they spoke about LLL completely overlap. Control variables included but not reported: Factor analytic household wealth index, whether household head is a man (=1), household's caste (schedule caste, schedule tribe, other backward category, or upper caste), man's age, man's education, woman's age, woman's education, and whether the woman is a farmer. Whether a man (woman) has an LLL lottery winner in his (her) agricultural network is used as an instrument for whether a man (woman) has an LLL adopter in his (her) agricultural network. * $p < 0.10$, ** $p < 0.05$, *** $p < 0.01$.

Table E2: Network Effects, Based on Water-Saving Benefits from Adoption

	Endline WTP	Baseline WTP
Man is linked to at least one water-saving adopter	145.582**	-50.596
	(57.803)	(38.118)
Man is linked to at least one non-water-saving adopter	0.739	-9.741
	(73.028)	(24.804)
Woman is linked to at least one water-saving adopter	4.362	19.911
	(47.215)	(30.327)
Woman is linked to at least one non-water-saving adopter	-97.192*	41.097
	(52.725)	(37.273)
One water-saving qualifying farmer in man's network	-22.900	18.507
	(30.246)	(25.161)
Two water-saving qualifying farmers in man's network	-107.684	-0.975
	(75.435)	(51.285)
Three water-saving qualifying farmers in man's network		-260.051*
		(146.649)
Four or more water-saving qualifying farmers in man's network	330.043*	-174.426
	(181.214)	(170.760)
One non-water-saving qualifying farmer in man's network	51.360	5.658
	(39.953)	(24.293)
Two non-water-saving qualifying farmer in man's network	92.445	-73.275
	(101.732)	(48.496)
Four or more non-water-saving qualifying farmer in man's network	243.094	-136.874
	(149.512)	(109.482)
One water-saving qualifying farmer in woman's network	32.785	-36.352*
	(37.557)	(20.353)
Two water-saving qualifying farmer in woman's network	66.385	-53.446*
	(48.242)	(31.576)
Three water-saving qualifying farmer in woman's network	110.022*	114.344
	(57.548)	(97.873)
Four or more water-saving qualifying farmer in woman's network	11.934	-64.826
	(111.596)	(63.914)
One non-water-saving qualifying farmer in woman's network	44.463	-19.142
	(43.545)	(33.582)
Two non-water-saving qualifying farmer in woman's network	-88.327*	1.075
	(45.523)	(65.029)
Three non-water-saving qualifying farmer in woman's network	-38.828	-82.210*
	(76.645)	(46.191)
Four or more non-water-saving qualifying farmer in woman's network	103.292	-97.636*
	(95.997)	(58.757)
Man's network size	-14.896*	14.812**
	(8.254)	(7.353)
Woman's network size	-0.643	0.390
	(4.140)	(1.975)
Baseline WTP	0.346***	
	(0.070)	
Constant	245.555***	25.510
	(52.879)	(34.684)
Observations	305	380
R^2	0.162	0.635
Controls	YES	YES
$\chi^2(1) : \beta_{msave} = \beta_{wsave}$	7.426***	2.107
$\chi^2(1) : \beta_{mnosave} = \beta_{wnosave}$	1.057	1.067

Standard errors in parentheses clustered at village-level. Control variables included but not reported: Factor analytic household wealth index, whether household head is a man (=1), household's caste (schedule caste, schedule tribe, other backward category, or upper caste), man's age, man's education, woman's age, woman's education, and whether the woman is a farmer. In the placebo regression with baseline WTP as the outcome variable, we also include whether the household was an auction winner in the regression specification. Whether a man (woman) has an LLL water-saving (non water-saving) lottery winner in his (her) agricultural network is used as an instrument for whether a man (woman) has an LLL water-saving (non water-saving) adopter in his (her) agricultural network. * $p < 0.10$, ** $p < 0.05$, *** $p < 0.01$.

Table E3: Gendered Network Effects, Based on Auction Discussion

PANEL A	
	Endline WTP
Man linked to at least one adopter	89.277
	(64.701)
Woman linked to at least one adopter	-35.121
	(65.290)
Man linked to adopter and discussed LLL	-3.932
	(85.204)
Woman linked to adopter and discussed LLL	-79.657
	(80.885)
Whether household discussed LLL (=1)	83.241**
	(32.676)
Man's network size	-1.033
	(7.773)
Woman's network size	-0.850
	(3.728)
Baseline WTP	0.306***
	(0.076)
Constant	199.562***
	(56.713)
Observations	280
R^2	0.197
Controls	YES

PANEL B		
	Men	Women
No Discussion	89.277	-35.121
	(65.476)	(65.290)
Discussion	85.35	-114.778***
	(56.664)	(40.386)

Standard errors in parentheses clustered at village-level. Network variables included but not reported: Whether the man has one, two, three, four or more qualifying farmers in his network; whether the woman has one, two, three, four, or more qualifying farmers in her network; whether the man has one, two, three, four or more qualifying farmers in his network and discussed LLL in the household; and whether the woman has one, two, three, four or more qualifying farmers in her network and discussed LLL in the household. Omitted category is whether the man (woman) has qualifying farmers and did not discuss LLL in the household. Non-network control variables included but not reported: Factor analytic household wealth index, household's caste (schedule caste, schedule tribe, other backward category, or upper caste), whether household head is a man (=1), man's age, man's education, woman's age, woman's education, and whether the woman is a farmer. Whether an individual has an LLL lottery winner (and LLL lottery winner and discussed LLL) in their agricultural network is used as an instrument for whether the individual has an LLL adopter (or LLL adopter and discussed LLL) in their agricultural network. * $p < 0.10$, ** $p < 0.05$, *** $p < 0.01$.

Table E4: Gendered Network Effects, By Type of Non-Adopters

Outcome variable: Endline WTP	Lottery Losers (1)	Auction Losers (2)
Man linked to at least one adopter	39.676	99.889
	(95.800)	(62.886)
Woman linked to at least one adopter	-17.608	-88.863***
	(79.170)	(29.838)
One qualifying farmer in man's network	38.531	-29.503
	(62.303)	(38.818)
Two qualifying farmers in man's network	34.935	-139.792*
	(108.314)	(77.976)
Three qualifying farmers in man's network	103.069*	-192.057
	(54.034)	(180.003)
Four or more qualifying farmers in man's network	170.040	-268.282
	(231.742)	(194.502)
One qualifying farmer in woman's network 57.801*		27.008
	(80.835)	(33.086)
Two qualifying farmers in woman's network	60.409	8.975
	(53.286)	(50.784)
Three qualifying farmers in woman's network	-36.666	-1.942
	(91.331)	(85.245)
Four or more qualifying farmers in woman's network	-110.792	255.060***
	(105.327)	(74.770)
Man's network size	-7.570	13.974
	(11.443)	(11.742)
Woman's network size	4.291***	-8.622***
	(1.425)	(3.193)
Baseline WTP	0.198	0.148
	(0.205)	(0.152)
Constant	274.025***	292.053***
	(93.145)	(92.504)
Observations	127	157
R^2	0.151	0.144
Controls	YES	YES
$\chi^2(1) : \beta_m = \beta_w$	0.351	6.848***

Standard errors in parentheses clustered at village-level. The regression in Column (1) uses the sub-sample of lottery losers, who qualified for but did not win the lottery. Their WTP was greater than or equal to the randomly-drawn binding price. The regression in Column (2) uses the sub-sample of auction losers, who had bid prices below the randomly drawn binding price and did not qualify for the lottery. Control variables included but not reported: Factor analytic household wealth index, whether household head is a man (=1), household's caste (schedule caste, schedule tribe, other backward category, or upper caste), man's age, man's education, woman's age, woman's education, and whether the woman is a farmer. Whether a man (woman) has an LLL lottery winner in his (her) agricultural network is used as an instrument for whether a man (woman) has an LLL adopter in his (her) agricultural network. * $p < 0.10$, ** $p < 0.05$, *** $p < 0.01$.

Table E5: Network Effects, Based on Different LLL Prices

	WTP =			
	250	350	450	600
Man linked to at least one adopter (β_m)	0.128	0.278***	0.068	0.062
	(0.081)	(0.072)	(0.092)	(0.082)
Woman linked to at least one adopter (β_w)	-0.084	-0.325**	-0.118	-0.027
	(0.052)	(0.129)	(0.124)	(0.057)
One qualifying farmer in man's network	-0.041	-0.040	0.017	-0.014
	(0.071)	(0.073)	(0.080)	(0.060)
Two qualifying farmers in man's network	-0.118	-0.105	-0.021	-0.178*
	(0.088)	(0.117)	(0.147)	(0.099)
Three qualifying farmers in man's network	-0.053	0.188	-0.203*	-0.128
	(0.228)	(0.277)	(0.115)	(0.090)
Four or more qualifying farmers in man's network	0.205	-0.283	-0.206	-0.335
	(0.238)	(0.486)	(0.406)	(0.336)
One qualifying farmer in woman's network	0.109*	0.212*	0.052	0.012
	(0.056)	(0.124)	(0.081)	(0.056)
Two qualifying farmers in woman's network	0.053	0.138	0.102	-0.030
	(0.084)	(0.122)	(0.088)	(0.067)
Three qualifying farmers in woman's network	-0.064	0.032	-0.126	-0.064
	(0.212)	(0.227)	(0.138)	(0.061)
Four or more qualifying farmers in woman's network	0.434***	0.020	-0.096	-0.026
	(0.161)	(0.243)	(0.172)	(0.068)
Man's network size	-0.011	0.015	0.005	0.018
	(0.013)	(0.027)	(0.024)	(0.021)
Woman's network size	-0.014*	0.009	0.007	0.001
	(0.009)	(0.009)	(0.006)	(0.003)
Baseline WTP	0.000***	0.001***	0.000**	0.000***
	(0.000)	(0.000)	(0.000)	(0.000)
Constant	0.732***	0.452***	0.126	-0.037
	(0.122)	(0.148)	(0.143)	(0.073)
Observations	305	305	305	305
R^2	0.105	0.144	0.072	0.082
Controls	YES	YES	YES	YES
$\chi^2(1) : \beta_m = \beta_w$	5.874**	17.97***	2.309	1.366

Standard errors in parentheses clustered at village-level. Control variables included but not reported: Factor analytic household wealth index, whether household head is a man (=1), household's caste (schedule caste, schedule tribe, other backward category, or upper caste), man's age, man's education, woman's age, woman's education, and whether the woman is a farmer. Whether a man (woman) has an LLL lottery winner in his (her) agricultural network is used as an instrument for whether a man (woman) has an LLL adopter in his (her) agricultural network. * $p < 0.10$, ** $p < 0.05$, *** $p < 0.01$.

Appendix F. Heterogeneity in Gendered Network Effects

Table F1: Household's Diesel Cost

PANEL A	
	Endline WTP
Man linked to at least one adopter	-77.907
	(110.560)
Woman linked to at least one adopter	-189.355**
	(86.518)
Man linked to adopter and has high diesel cost	436.892*
	(241.087)
Woman linked to adopter and has high diesel cost	191.369
	(162.061)
Whether household has high diesel cost	27.791
	(27.880)
Man's network size	-12.125
	(10.793)
Woman's network size	4.415***
	(1.045)
Baseline WTP	0.138
	(0.167)
Constant	289.713***
	(80.909)
Observations	146
R^2	0.188
Controls	YES

PANEL B		
	Men	Women
Low Diesel	-77.907	-189.355**
	(110.560)	(86.518)
High Diesel	358.985	2.013
	(245.057)	(116.351)

Standard errors in parentheses clustered at village-level. Households that had a diesel cost per acre greater than or equal to the sample mean were classified as incurring a high diesel cost. We have diesel-use data for 221 households, and 38 percent of them were classified as high diesel use households. Network variables included but not reported: Whether the man has one, two, three, four or more qualifying farmers in his network; whether the woman has one, two, three, four, or more qualifying farmers in her network; whether the man has one, two, three, four or more qualifying farmers in his network and had high diesel cost; and whether the woman has one, two, three, four or more qualifying farmers in her network and had high diesel cost. Omitted category is whether the man (woman) has qualifying farmers and has low diesel cost. Non-network control variables included but not reported: Factor analytic household wealth index, household's caste (schedule caste, schedule tribe, other backward category, or upper caste), whether household head is a man (=1), man's age, man's education, woman's age, woman's education, and whether the woman is a farmer. Whether an individual has an LLL lottery winner (along with its interaction with whether the household has high diesel cost) in their agricultural network is used as an instrument for whether the individual has an LLL adopter (or LLL adopter exhibiting that trait) in their agricultural network. * $p < 0.10$, ** $p < 0.05$, *** $p < 0.01$.

Table F2: Household Wealth

PANEL A	
	Endline WTP
Man linked to at least one adopter	129.080*
	(68.771)
Woman linked to at least one adopter	-231.657***
	(72.125)
Man linked to adopter and is classified poor	-101.092
	(101.444)
Woman linked to adopter and is classified poor	194.036**
	(96.303)
Whether household is poor (=1)	-81.628***
	(29.950)
Man's network size	-7.173
	(7.967)
Woman's network size	-2.217
	(3.710)
Baseline WTP	0.334***
	(0.067)
Constant	307.731***
	(62.763)
Observations	305
R^2	0.199
Controls	YES

PANEL B		
	Men	Women
Non-Poor	129.080*	-231.657***
	(68.771)	(72.125)
Poor	27.988	-37.621
	(58.228)	(42.714)

Standard errors in parentheses clustered at village-level. Households with wealth index below the sample mean (=0) are classified as poor. 65 percent households were classified as poor. Network variables included but not reported: Whether the man has one, two, three, four or more qualifying farmers in his network; whether the woman has one, two, three, four, or more qualifying farmers in her network; whether the man has one, two, three, four or more qualifying farmers in his network and is classified as poor; and whether the woman has one, two, three, four or more qualifying farmers in her network and is classified as poor. Omitted category is whether the man (woman) has qualifying farmers and is non-poor. Non-network control variables included but not reported: Factor analytic household wealth index, household's caste (schedule caste, schedule tribe, other backward category, or upper caste), whether household head is a man (=1), man's age, man's education, woman's age, woman's education, and whether the woman is a farmer. Whether an individual has an LLL lottery winner (along with its interaction with whether the household is classified as poor) in their agricultural network is used as an instrument for whether the individual has an LLL adopter (or LLL adopter exhibiting that trait) in their agricultural network. * $p < 0.10$, ** $p < 0.05$, *** $p < 0.01$.

Table F3: Woman's Opinion is Valued by the Man

PANEL A		
	Woman's Report	Man's Report
Man linked to at least one adopter	72.067**	77.456*
	(36.728)	(42.278)
Woman linked to at least one adopter	19.039	-96.646**
	(80.276)	(48.322)
Woman linked to one adopter and perceives man values opinion	-118.012	
	(86.300)	
Woman's opinion is valued in the household	33.505	
	(39.211)	
Woman linked to one adopter and husband values opinion		7.159
		(68.036)
Husband values woman's opinion		44.399**
		(21.448)
Man's network size	-0.341	0.492
	(6.772)	(6.963)
Woman's network size	-2.776	-2.879
	(4.057)	(4.000)
Baseline WTP	0.297***	0.309***
	(0.061)	(0.066)
Constant	233.980***	216.250***
	(54.471)	(54.222)
Observations	297	305
R^2	0.134	0.163
Controls	YES	YES

PANEL B		
	Men	Women
Woman reports her technology opinion is valued[c]	72.067**	-98.973***
	(36.728)	(27.989)
Woman reports her technology opinion is not valued[c]	72.067**	19.039
	(36.728)	(80.276)
Man reports he values wife's opinion[d]	77.456*	-89.487**
	(42.278)	(39.967)
Man reports he does not value wife's opinion[d]	77.456*	-96.646**
	(42.278)	(48.322)

Standard errors in parentheses clustered at village-level. In our sample, 66 percent women reported that their husband valued their opinion about agricultural technologies and 67 percent men reported that they valued their wives' opinion about agricultural technologies. Network variables included but not reported: Whether the man has one, two, three, four or more qualifying farmers in his network; whether the woman has one, two, three, four, or more qualifying farmers in her network; whether the man has one, two, three, four or more qualifying farmers in his network and woman's opinion is valued in the household; and whether the woman has one, two, three, four or more qualifying farmers in her network and woman's opinion is valued in the household. Omitted category is whether the man (woman) has qualifying farmers and is non-poor. Non-network control variables included but not reported: Factor analytic household wealth index, household's caste (schedule caste, schedule tribe, other backward category, or upper caste), whether household head is a man (=1), man's age, man's education, woman's age, woman's education, and whether the woman is a farmer. Whether an individual has an LLL lottery winner (along with its interaction with whether the woman's opinion is valued in the household) in their agricultural network is used as an instrument for whether the individual has an LLL adopter (or LLL adopter exhibiting that trait) in their agricultural network. * $p < 0.10$, ** $p < 0.05$, *** $p < 0.01$.

References

Barrett, C.B., and M.R. Carter. 2013. “The economics of poverty traps and persistent poverty: Empirical and policy implications.” *Journal of Development Studies* 49:976–990.

Fafchamps, M., and F. Gubert. 2007. “The formation of risk sharing networks.” *Journal of Development Economics* 83:326–350.

Maertens, A., and C.B. Barrett. 2013. “Measuring social networks’ effects on agricultural technology adoption.” *American Journal of Agricultural Economics* 95:353–359.

Santos, P., and C.B. Barrett. 2010. “Identity, interest and information search in a dynamic rural economy.” *World Development* 38:1788–1796.
